# Hydrogels as Drug Delivery Systems: A Review of Current Characterization and Evaluation Techniques

**DOI:** 10.3390/pharmaceutics12121188

**Published:** 2020-12-07

**Authors:** Margaux Vigata, Christoph Meinert, Dietmar W. Hutmacher, Nathalie Bock

**Affiliations:** 1 Institute of Health and Biomedical Innovation (IHBI), Queensland University of Technology (QUT), Kelvin Grove, QLD 4059, Australia; margaux.vigata@hdr.qut.edu.au (M.V.); christoph.meinert@qut.edu.au (C.M.); dietmar.hutmacher@qut.edu.au (D.W.H.); 2 School of Mechanical, Medical and Process Engineering, Science and Engineering Faculty (SEF), Queensland University of Technology (QUT), Brisbane, QLD 4059, Australia; 3 Herston Biofabrication Institute, Metro North Hospital and Health Services, Herston, QLD 4006, Australia; 4 School of Biomedical Sciences, Faculty of Health, Queensland University of Technology (QUT), Brisbane, QLD 4059, Australia; 5 Australian Research Council Industrial Transformation Training Centre in Additive Biomanufacturing, Queensland University of Technology (QUT), Brisbane, QLD 4059, Australia; 6 Translational Research Institute, Woolloongabba, QLD 4102, Australia

**Keywords:** hydrogel drug delivery system, characterization techniques, drug release kinetics, drug diffusion coefficient

## Abstract

Owing to their tunable properties, controllable degradation, and ability to protect labile drugs, hydrogels are increasingly investigated as local drug delivery systems. However, a lack of standardized methodologies used to characterize and evaluate drug release poses significant difficulties when comparing findings from different investigations, preventing an accurate assessment of systems. Here, we review the commonly used analytical techniques for drug detection and quantification from hydrogel delivery systems. The experimental conditions of drug release in saline solutions and their impact are discussed, along with the main mathematical and statistical approaches to characterize drug release profiles. We also review methods to determine drug diffusion coefficients and in vitro and in vivo models used to assess drug release and efficacy with the goal to provide guidelines and harmonized practices when investigating novel hydrogel drug delivery systems.

## 1. Introduction

The development of polymeric drug delivery systems as an alternative to conventional drug formulations has grown steadily for several decades now, mostly trying to address inadequate local drug availability and challenges associated with delivery sites. Thermoplastic- and in particular hydrogel-based scaffolds are attractive for controlled drug delivery as their properties can be tuned during manufacturing while also being amenable to safe implantation, release, and degradation [1]. Hydrogels are highly hydrated mesh networks formed from natural, synthetic, or semi-synthetic polymers, which are physically or covalently crosslinked [2]. This class of materials is used for local drug delivery because they provide high biocompatibility [3], drug protection [4], spatiotemporal control of the drug release [4], and physicochemical tailorability [5]. Furthermore, hydrogels enable the encapsulation and the delivery of drugs spanning a wide range of properties, including small molecules [6,7], proteins [8,9], and nucleic acids [10,11]. Hydrogels and hydrogel drug delivery systems are traditionally defined as being natural or synthetic. Hydrogels of natural origin include; chitosan [12], alginate [13], fibrin [14], gelatin [15], or hyaluronic acid-based hydrogels [7]; while poly(ethylene glycol) (PEG) [16] or poly(vinyl alcohol) [17], are common synthetic hydrogels. There is also a third category that englobes the semi-synthetic hydrogels like gelatin methacryloyl hydrogels, which are gelatin-based but were functionalized by synthetic methacryloyl groups [5]. To date, hydrogel systems have been extensively studied to deliver drugs for the treatment of cancer [13] and infections [18], wound healing [19], and for tissue engineering [7] applications. Hydrogels are also versatile drug delivery systems and as such present the advantage of being amenable to various administration routes [20]. They were applied to the enteral/oral administration route [12,21], local parenteral route, which refers to local in situ implantation [22,23], but also the topical/transdermal route or the ocular route that are usually more relevant for drug delivery in two dimensions [24,25,26,27].

In this review, we define hydrogel drug delivery systems as systems for drug delivery in three dimensions that are destined to be administered via the oral/enteral route or the parenteral route that largely means local implantation of the hydrogel inside the body.

Various work focusing on the material and structural characterization techniques and methods for hydrogel drug delivery systems have been previously reported and reviewed [28,29,30,31]. However, some general guidelines are needed to understand better which characterizations should be used for different types of systems. Standardization of methods will further enable improved correlation *of* in vitro and in vivo release kinetics with mechanisms of action—critical to ensuring system efficiency and effective path towards clinical translation.

Appropriate drug quantification is the first step to drug delivery system validation. However, drug detection techniques present limitations, such as inadequate detection limits [32,33], cost, complexity, sensitivity, and selectivity [34], which need to be balanced adequately with each type of system in order to match the right system with the right method. In parallel, release assay protocols are also important to validate new systems, yet present with a myriad of parameters (including agitation [35], temperature [36], pH [12], volume [33], and composition [37] of the release media) used inconsistently between research groups without strong apparent rationale. This lack of standardization leads to the limited prediction of in vivo release behavior, lack of reproducibility of results, and thus, by extension, lack of translatability [33]. Drug diffusion coefficients are other important predictors of release behavior, yet too often excluded. This arises partly because its empirical measurement is challenging, requiring sophisticated and complex techniques and experimental setups, such as nuclear magnetic resonance [38], fluorescence microscopy [39,40], and microfluidic platforms [41].

Finally, in addition to physicochemical characterization of drug release in cell-free environments, in vitro models are typically used to validate and further assess the biocompatibility and efficacy of drug release in a biological context. Yet, simple 2D cell culture systems are often used, although more complex 3D microenvironments, which have now become the gold standard for cell culture in other areas of research such as cancer, would be one step closer to the physiological situation [42]. Similarly, preclinical studies of hydrogel drug delivery systems are a milestone in the evaluation of the translatability of a system. Nevertheless, most in vivo studies focus on drug efficacy measured by indirect methods, such as the tumor volume monitoring or immunohistochemistry staining of the tissues after sacrifice [15,43,44]. In vivo drug release is not routinely monitored, representing a failure in providing a complete assessment of actual pharmacokinetics.

In summary, in order to address the shortcomings highlighted above, this review provides on outlook on the characterization and evaluation techniques for hydrogel drug delivery systems by defining; (1) techniques used for drug detection and quantification; (2) guidelines to interpret release kinetics; (3) drug diffusion coefficient evaluation methods, and (4) design of relevant in vitro/in vivo experiments to assess efficacy. Our aim is to provide guidance to design more comprehensive and impactful investigations that support the development of hydrogel drug delivery systems that are better characterized, leading, ultimately, to better clinical translation.

## 2. Drug Detection and Quantification

The choice of an appropriate method for the detection and quantification of drugs released from hydrogel delivery systems primarily depends on the drug type, but convenience, complexity, cost, selectivity, and sensitivity of the technique should be considered. Hydrogel-drug delivery systems rely on the same analytical technologies from the most inexpensive and most practical optical spectroscopy techniques [6,7,15,45,46], to the most selective, sensitive, and costly ones, like high performance liquid chromatography (HPLC) [43,47], mass spectrometry [48,49,50], or polymerase chain reaction (PCR) [10]. Table 1 summarizes the advantages and disadvantages of the techniques that will be discussed in this section.

### 2.1. Optical Spectroscopy

#### 2.1.1. Molecular Absorption

Molecular absorption spectroscopy allows the detection and quantification of any molecule absorbing in the Ultraviolet and visible light spectrum (UV-Vis) [51,52] (Table 1). It is based on the Beer–Lambert law:

(1) A=εlc

where *ε* is the molar attenuation coefficient (L/mol/cm), *l* is the optical path length (cm), and *c* the concentration of the substance analyzed (mol/L) [51,52].

Ultraviolet light (100 to 400 nm) or visible light (400 to 700 nm) is emitted upon the object to be analyzed, i.e., the drug-containing solution. When the emitted light passes through the sample, the molecules in the solution absorb photons at specific wavelengths according to the molecule structure, functional groups, and conjugations. Quantification is obtained through the ratio of the intensities from the emitted light versus the emitted light from a blank sample. This ratio is directly dependent on the concentration of molecules of interest present in the solution [53,54,55,56].

As shown in Table 2, the ultraviolet-visible spectroscopy (UV-Vis) method is widely used to quantify small-molecule drugs because of its convenience and cost-effectiveness. However, the method is relatively insensitive and, therefore, inadequate for low concentration samples, usually limited at around 0.1–0.2 mg/mL of drug concentration depending on the drug [6,57]. Another disadvantage of UV-Vis spectroscopy is the lack of specificity. Zhang et al. developed a hydrogel from xanthan gum to release bovine serum albumin (BSA) and 5-fluorouracil, which were detected using UV-Vis at 280 nm and 286 nm for BSA and 5-fluorouracil, respectively [58]. Here, the detection wavelengths are very similar, thus compromising the selective detection of the compounds.

There are colorimetric assays for protein detection that use light absorbance, one of which is the Biuret method that relies on the principle that proteins and amino acids form a colored complex with copper, which is detectable at 540 nm [73,74]. The Bradford and Lowry protein detection assays were developed later to improve the Biuret method. The Bradford method relies on the complexation of proteins with coomassie dye, whereas the Lowry test quantifies the Cu-protein complex formation that provides a Cu^2+^ ion, which can be reduced and detected via absorbance [75,76]. Both methods are more sensitive than the Biuret test [77]. Another assay, which is as sensitive as the Lowry test but easier to implement and available in a commercial kit, is the bicinchoninic acid assay (BCA assay). In the BCA assay, peptide bonds in proteins reduce Cu^2+^ into Cu^+^ and, in turn, the complex formed by Cu^+^ and the bicinchoninic acid form a color that can be detected via absorbance and directly correlated with the protein concentration [78,79]. Colorimetric assays are more specific than direct spectrophotometry for protein detection. Moreover, the BCA, Lowry, and the Bradford tests are often more sensitive than direct spectrophotometry, but both spectrophotometry and colorimetric options are limited by their range of detection. More specifically, a study found that the lower limit of detection of pure BSA was 0.391 mg/mL for the Biuret test, 0.012 mg/mL for the Lowry test, 0.006 mg/mL for the Bradford test, 0.024 mg/mL for the BCA assay, and finally 0.049 mg/mL for the direct spectrophotometry at 280 nm [32].

UV-VIS direct spectrophotometry and colorimetric methods are fast and inexpensive methods to investigate drug release from a hydrogel delivery system [32]; however, the detection of concentrations below 1 µg/mL requires more sophisticated detection methods. Besides, the UV-VIS method will detect any molecule that absorbs at the chosen wavelength, thus is not selective [53].

#### 2.1.2. Molecular Emission

Contrary to the UV-VIS method, fluorescence detection relies on the absorption of a high energy light (excitation) of the molecule, which then emits light of lower energy that is detected [80]. Some molecules and drugs inherently possess fluorescent properties, which have been used in the drug delivery field to detect and image the drug or the drug-loaded delivery system [81]. One common example is Doxorubicin, as it can be imaged using its native fluorescence (excitation at 470 nm; emission at 595 nm), and it is a very potent and commonly used anticancer drug [82,83]. Doxorubicin was investigated for its native fluorescence properties and was detected and quantified in 

phosphate-buffered saline (PBS) [84,85] and within cells, in vitro, to observe the cellular uptake of the drug [86,87]. Doxorubicin was previously used as a guiding imaging agent to achieve co-delivery of two agents in a drug delivery system with dual fluorescence enabling imaging-guided chemotherapy providing in vitro and in vivo real-time tracking [88].

However, doxorubicin remains an exception, and drug detection via fluorescence usually requires fluorescent tagging. The versatility of the molecular emission spectroscopy technique enables the detection of small molecules, proteins, polysaccharides, and oligonucleotides (Table 3).

Fluorescence detection and quantification has been used for small molecules [15] but also to label proteins [8]. The model protein BSA can be FITC-labelled to be detected via fluorescence intensity (493 nm). A FITC-BSA quantification used a calibration curve with a lower limit of detection in the order of ng/mL (minimum 0.005 µg/mL to a maximum of 1.9 µg/mL) [8]. The sensitivity of fluorescence molecule detection is indeed superior and around 1000-fold higher than that of UV-VIS absorption. Detection kits can also aid the quantification of RNA biomolecules. Such kits were used to quantify siRNA molecules released from chitosan hydrogels by fluorescence detection (BLOCK-iT Fluorescent Oligo, Life Technologies) [93] or plasmid DNA (pDNA) [11].

**Figure 1:**
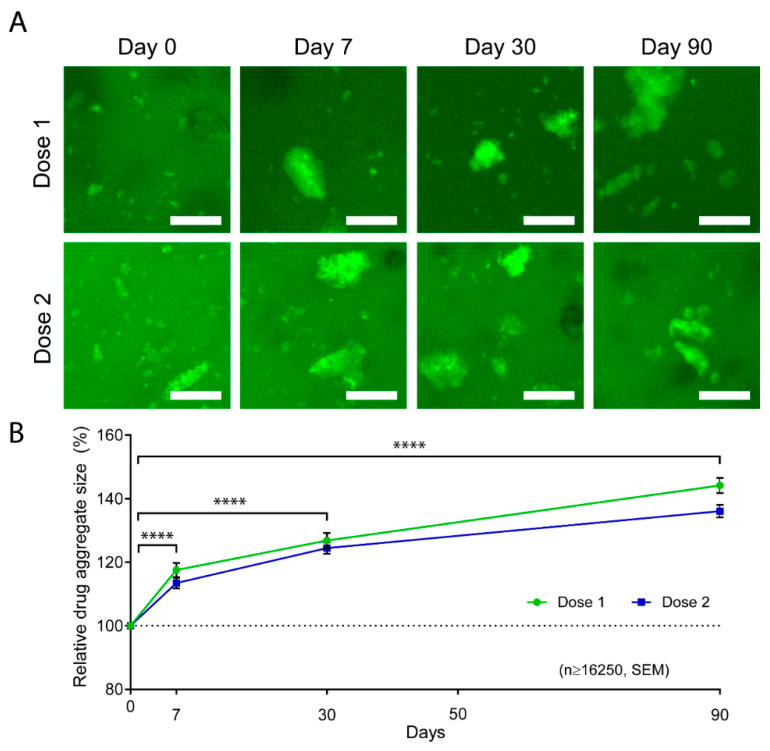
Abraxane^®^ contained in gelatin methacryloyl (GelMA) hydrogels imaging during a release assay. Fluorescein-5-Isothiocyanate (FITC) was used to label the albumin part of Abraxane^®^ and image aggregates retained in 10% GelMA hydrogels at day 0, 7, 30, and 90 of a release in Phosphate-Buffered Saline (PBS). (**A**) Representative z-stack maximum projections of FITC-Abraxane^®^ in 10% GelMA. Dose 1 = 37.5 µg of Abraxane^®^; Dose 2 = 75 µg of Abraxane^®^. Scale bar = 25 µm. (**B**) FITC-Abraxane^®^ aggregate size normalized to day 0. Data are shown as means ± standard error of the means, *n* ≥ 16,250 aggregates, **** *p* < 0.0001. Reproduced from [94] under a CC BY 4.0 license.

Fluorescence is a precious tool for drug detection that is more sensitive and selective than UV-Vis and allows the precise quantification of proteins and oligonucleotides. It is also used for imaging and visualizing the drug or drug complex within the hydrogel drug delivery system (Figure 1A), which is useful to quantify aggregate size (Figure 1B), as we did in our previous work with FITC-Abraxane^®^ in GelMA hydrogels (Figure 1) [94], or to monitor the drug diffusion coefficient through the hydrogel. The later application will be further discussed in the section on drug diffusion (Section 4).

#### 2.1.3. Enzyme-Linked Immunosorbent Assay

In the field of protein delivery, particularly growth factor delivery, an enzyme-linked immunosorbent assay (ELISA) is often used to detect and quantify the protein of interest selectively with lower limits of detection in the range of pg/mL depending on the protein and the commercial kit used [95]. ELISA is based on antibodies that will selectively bind to the protein of interest. The detection is performed via a reporter enzyme linked to a primary or secondary antibody, which becomes optically active when its substrate is introduced in the reactive solution, thus can be detected via fluorescence or colorimetry spectroscopy. ELISA was used to detect and quantify the expression of vascular endothelial growth factor (VEGF) after the pDNA coding for VEGF release from hydrogel drug delivery systems [10], as well as stromal cell-derived factor 1 (SDF1) protein [46]. The DuoSet^®^ Human VEGF ELISA kit was used to quantify its cumulative release from a fibrin hydrogel delivery system where the total drug cargo was 2 µg; thus, the technique was used to quantify concentrations below 1 µg/mL [14]. The selectivity of ELISA detection was also reported to range between 0 and 4000 pg/mL for the detection of platelet-derived growth factor-BB delivered by PEG hydrogels [96]. ELISA can also be modified to detect oligonucleotides and was used to evaluate the penetration in the skin of DNAzymes delivered by a chitosan hydrogel (hybridization-ELISA) [97].

While showing excellent advantages in selectivity and sensitivity for protein detection and quantification, the detection by ELISA can be impaired by interferences [98]. For instance, Bock et al. compared cell-related assays with ELISA assays to demonstrate the limitations of ELISA in providing accurate measurements of GF concentrations, which almost always measured a non-complete release of growth factors from a polyester-growth factor (GF) deliver system. This was hypothesized to be due to GF detection issues (aggregation during ELISA assay) since full cargo was released and the system performed according to the full amount of drugs loaded, as assessed by cell release assays [98]. Ultimately, ELISA remains a highly selective and sensitive method, easy to perform and suitable to detect a target protein in various liquid types, e.g., plasma, tissue, and cellular extracts, or urine, among others. Nevertheless, we advise correlating ELISA results with a second detection method to measure protein bioactivity following release.

### 2.2. High Performance Liquid Chromatography

High-performance liquid chromatography (HPLC) is used to separate and concentrate the sample before quantification by UV absorbance, and less frequently fluorescence spectrometry [99], or mass spectrometry. Separation of therapeutic molecules based on their physicochemical properties is performed using two non-miscible phases, a static and a mobile phase, which are chosen depending on the target molecule [100]. The utilization of HPLC allows for selective and sensitive concentration of target molecules [101], but requires the development and optimization of the HPLC method, which is time-consuming. Additionally, HPLC requires an extraction step either to extract a hydrophobic drug into an appropriate solvent (liquid-liquid separation) [43,102,103], or to remove any impurities, e.g., plasma protein, or potential degradation products from the hydrogels, from the samples (solid-liquid separation) [47,97,104].

The hydrophobic anticancer drug paclitaxel was quantified using HPLC-UV at 273 nm to quantify its release from a Pinus koraiensis polysaccharide-based hydrogel drug delivery system in PBS [43]. Because of the hydrophobic nature of paclitaxel, a liquid-liquid extraction step was added between the sampling and the HPLC-UV analysis. The release medium was lyophilized; then, paclitaxel was solubilized in acetonitrile. Then, a centrifugation step removed insoluble particles, and the supernatant was injected in the HPLC-UV for quantification [43]. Zhao et al. also used HPLC to quantify paclitaxel from their PLGA nanoparticle embedded in a photopolymerizable polyethylene glycol dimethacrylate (PEG-DMA) hydrogel to treat Glioblastoma multiforme in vivo [47].

HPLC is a very efficient analytical tool that provides high sensitivity that enables a lower limit of detection in the order of ng/mL depending on the molecule and method developed [105], but also requires complex technical skills and is costly and time-demanding [34].

### 2.3. Mass Spectroscopy

Mass spectrometry is an analytical technique that detects and quantifies molecules with a high degree of sensitivity and selectivity. It can detect small molecules, as well as peptides, proteins, oligosaccharides, and DNA fragments [106]. The technique is based on the fragmentation of the molecule of interest into ions that are characteristics of that molecule since every molecule has a unique fragmentation pattern. The ratio of the mass and charge of the detected ion is determined, and thus the technique provides as outputs a retention time and a mass/charge spectra for the target molecule that is used, for comparison with a standard to provide a definite quantification [107]. Mass spectrometry is usually coupled with a molecule separation and concentration unit like HPLC. Then, the separated compounds enter the ion source where they are ionized via electrospray ionization (ESI) [108] or matrix-assisted laser desorption/ionization (MALDI) [109,110]. The ion(s) mass and charge are detected and quantified. The other advantage of the technique is that it is high throughput owing to embedded autosampling in most current machines.

This technique is the most selective and sensitive and can detect a wide range of molecules. Yet, its usage remains limited in the drug hydrogel delivery field [48,49,50] because the technique requires the appropriate expertise to develop and validate the method as well as being expensive [111].

### 2.4. Quantitative Polymerase Chain Reaction

The polymerase chain reaction is the gold standard technique for the detection of DNA [112]. The technique is composed of three steps: DNA denaturation, annealation, and extension. Double-stranded DNA is denatured to obtain single-stranded DNA. Then in the annealing phase, the forward and reverse primer hybridize specifically and selectively to a target sequence of the DNA molecule. In the third and last phase, a DNA polymerase enzyme carries out the extension of the primer-target DNA complex. In quantitative PCR (qPCR), also known as real-time qPCR, the quantity of DNA present at each cycle is measured via a fluorescence signal coming either from a specific hydrolysis probe or a non-specific dye that binds to double-stranded DNA following the extension step. This technique is inherently sensitive and very effective but also quite expensive. Real-time (RT)-qPCR was used by Peng et al., who developed a hydrogel to co-deliver resveratrol and a plasmid DNA coding for the VEGF growth factor for the application of wound healing [10]. They characterized the expression of VEGF as a protein in vitro via ELISA assay, but, in vivo, they used RT-qPCR to detect and quantify the presence of mRNA encoding for VEGF. They found that the drug-treated groups were expressing more VEGF compared to the untreated groups and that the increase was incremental over time [10]. This was an indirect way of measuring the release of plasmid DNA by measuring its translation in mRNA hence its efficacy but not an absolute quantitation [10].

## 3. Release Assays and Interpretation of Release Kinetics

**Figure 2:**
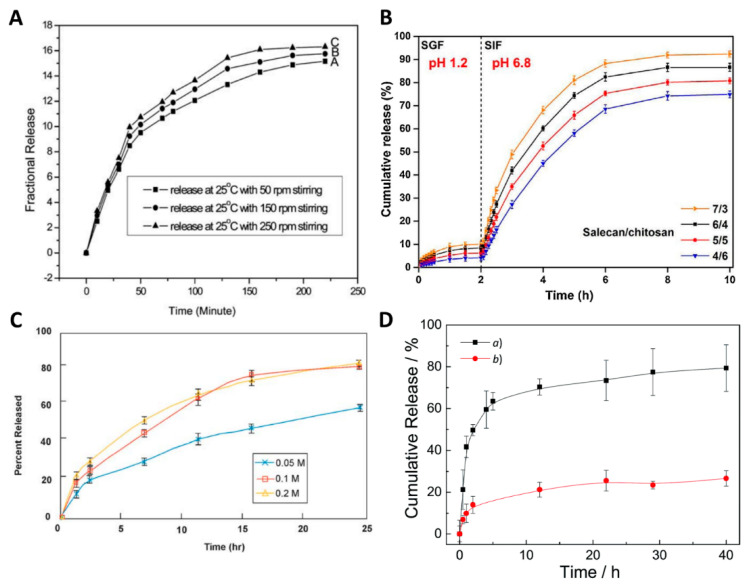
Parameters influencing in vitro drug release. (**A**) Release of indomethacin from a hydrogel system at 25 °C with different stirring rates (50 rpm, 150 rpm, 250 rpm). Reproduced with permission from [35]. Copyright the Royal Society of Chemistry, 2010. (**B**) Cumulative release (%) of Vitamin C from Salecan/chitosan polyelectrolyte complex hydrogels at different hydrogel ratio in simulated gastric fluid (SGF) for the first two hours and simulated intestinal fluid (SIF) from the second hour until the end of the release assay. Reproduced with permission from [12]. Copyright Elsevier, 2020. (**C**) Release of Verapamil in phosphate buffers at different molarities (0.05 M, 0.1 M, and 0.2 M). Data are shown in means ± standard deviation, *n* = 6. Reproduced with permission from [113]. Copyright Dissolution Technologies, 2007. (**D**) In vitro release of Bufalin at 10 mM PBS, pH 7.4, 37 °C in the (**a**) presence, and (**b**) absence of esterase (10 units). Reproduced with permission from [37]. Copyright the Royal Society of Chemistry, 2016.

Traditionally, hydrogel-drug delivery system samples are immersed in a releasing medium, partly mimicking physiological conditions in terms of pH (saline buffer) with control over temperature and CO_2_ concentration (using incubators) [8,19,61,68]. Agitation, ionic strength, enzymatic presence/absence are other parameters that are often controlled in the final release system [12,35,37,113]. At every time point, supernatant samples are collected for drug concentration analysis. Upon quantification, a cumulative release curve is typically drawn, which corresponds to the amount or percentage of drug released over time. Release profiles give valuable information and indication about the release kinetics, the amount released at different phases of the release, and clues about the release mechanisms [114]. The variety of polymers, drugs, release factors, and kinetics can, in theory, be tailored to a burst, controlled, biphasic, pulsatile, or sustained-release with zero-order kinetics [4]. Nevertheless, the choice of release method deeply affects the characterization of drug-hydrogel systems (Figure 2). Such assays need to be clearly rationalized and standardized for each drug-hydrogel system, ideally tailored according to the final application and validated in the context of the actual application. Subsequently, other tools are needed to analyze further the cumulative release curves, such as mathematical models that can be applied to the release data and which give an indication of the release mechanisms; and proper statistical analysis that can identify significant differences between the experimental groups and/or release parameters.

### 3.1. Drug Release Methods

To study release profiles and mechanisms in vitro, the rationale has been to simulate physiological conditions. Therefore, researchers mostly use phosphate-buffered saline (PBS) to immerse their gels because it has physiological pH (7.4) and osmolality, combined with the appropriate incubation at body temperature (37 °C) and agitation [8,19,61,68]. The previously mentioned parameters should be cautiously chosen as they have a significant impact on the release kinetics. Indeed, the agitation speed, the temperature, the pH, and the compositions of the release media are the most important to significantly impact the release (Figure 2).

#### 3.1.1. Agitation

Agitation typically increases the diffusive release of drugs from hydrogel drug delivery systems. By creating flow, drug mass transport between the hydrogel compartment and the release media compartment is favored. In the laboratory, agitation is typically applied by using a shaking incubator, shaking water bath or magnetic stirrer and is measured in rotations per minute (rpm) [33]. This effect was described by Han et al., who designed a protein nanocontainer from 12-hydroxystearic acid (HSA) gelator for indomethacin (a nonsteroidal anti-inflammatory drug). Figure 2A shows the in vitro release profiles obtained at room temperature and in PBS, and the authors studied the drug release under different agitation speeds, i.e., 50 rpm, 150 rpm, and 250 rpm. The authors stipulated that higher agitation speeds led to quicker drug release, although no statistical analysis was used and error bars were not presented, questioning whether any significance was achieved within this agitation range and this system [35]. The effect of agitation was, however, also demonstrated in other studies, enforcing the concerns about the critical role of this parameter, although not sufficiently studied yet in hydrogel systems [113,115,116,117]. Table 4 provides the parameters that have been used for the limited in vitro release studies from various hydrogel drug delivery systems. Overall, when agitation is applied, it is typically within the 50 to 150 rpm range. In general, we advise applying a continuous agitation speed of 100 rpm, which approximates flow velocities representative of the physiological flow in the bloodstream [33]. Higher agitation would accelerate the release and pose the risk of premature damage of the hydrogel system [33]. Hydrogel systems with large mesh sizes and small drug sizes would also be particularly impacted by the agitation parameter. The vessel size is also important because, for large vessels such as the United States Pharmacopoeia (USP) apparatus (900 mL), the agitation is performed by stirring paddles, while for smaller vessels, like small tubes containing 1 to 15 mL, the agitation has to be applied externally via a shaking incubator. Alternatively, a magnetic stirrer can be used for a medium-sized release vessel. To ensure reproducibility, both agitation speed and release vessel size must be considered together. However, most studies completely overlook the parameter, i.e., did not apply any agitation or applied agitation or do not specify the speed, which prevents any replication of the work, and thus a comparison of results [10,58,63,118].

#### 3.1.2. Temperature

Secondly, the temperature of the release medium is essential and should be the physiological temperature of the human body (37 °C) to ensure the relevance of the results and the best prediction of in vivo release. However, some studies failed to apply a temperature control to the experimental release setup and used the room temperature around 23–25 °C, which likely prevents accurate prediction of in vivo release (Table 4). In one study, however, N-tert-butylmaleimic acid-based hydrogel drug delivery systems were studied. Such types of gel are susceptible to temperature, which impacts their swelling behavior and, ultimately, the release kinetics [36]. Specifically, the release of rhodamine 6G from a hydrogel-drug delivery system made from Poly(N-isopropylacrylamide), p(NIPAM), and N-tert-butylmaleimic acid (TBMAC) monomers was performed both at physiological temperature and at room temperature [36]. A burst release was observed at 23 °C reaching 100% of release within 1–2 days, whereas, at 37 °C, the release was controlled and reached a maximum of around 40% after 70 h. The hydrogel system was indeed thermo-responsive and characterized by a hydrophilic molecular structure at room temperature while, at body temperature, it had transitioned from a hydrophilic coil structure allowing hydrogen bonds and water-based diffusion to a hydrophobic material that caused the hydrogel structure and pores to shrink, thereby retaining the drug [36]. The study, and some others [119,120,121], highlighted the impact of the temperature on release. Thus, while not all hydrogel systems may be affected by temperature, it is recommended to use physiological temperatures for drug release studies.

#### 3.1.3. Release Media Volume

**Figure 3:**
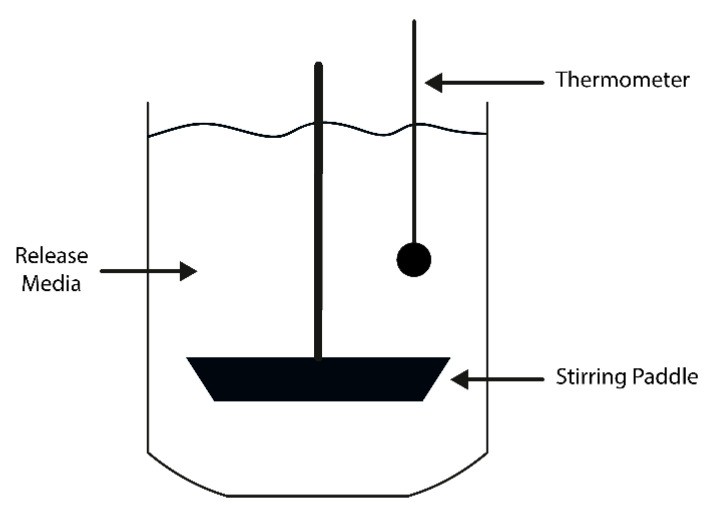
Schematic representation of the USP Apparatus 2. The temperature is closely controlled and monitored thanks to a thermometer. The release media can be homogeneously mixed with a stirring paddle.

Thirdly, the volume of release media also varies between studies from a few mL up to 900 mL (Table 4). The choice of volume to use also depends on the lower limit of detection of the chosen drug detection method, the solubility, and stability of the drug in the release media [33,122,123]. In addition, it is the ratio between the volume of release media and drug amount that is important as it controls the drug concentration range, which should be monitored to ensure drug solubility; perfect sink conditions, and drug detection by the chosen analytical detection method. Consequently, when upscaling the release media volume, the ratio between drug and release media to volume must be maintained for the same drug detection method. For example, using a high release media volume along with a USP^®^ dissolution apparatus, a semi-automated device composed of several glass vessels containing an agitation paddle and used to perform in vitro drug release assays in the pharmaceutical industry, has been used for hydrogel tablets destined for oral route and systemic absorption (Figure 3). The drug quantity per tablet is usually in the mg range, and, therefore, a large release volume, e.g., 900 mL, is used. This is the typical working volume of release assays that use USP^®^ dissolution apparatuses, as it ensures a homogeneous agitation. USP^®^ dissolution apparatuses provide accurate control of temperature and agitation speed [124] and may be a highly useful tool for higher-level characterization for higher volume hydrogel systems. Nevertheless, for other hydrogel drug delivery systems destined to be implanted and/or used to release a drug locally; often, the drug quantity per sample is in the sub-milligram range [94,125]. This could explain why the dissolution apparatus setup, however useful, may not be suitable to study the release of such smaller-scale systems [64,65].

At each time point, the volume of release media, which is sampled and refreshed, has an impact. In the reported literature, the sampled volume varies and is not always replaced by fresh media (Table 4). The intent of refreshing the release medium is to partly mimic the clearance system in vivo. Furthermore, such in vitro practice influences release profiles and, in many cases, maintains drug concentrations below 10% of the saturation concentration in the release media [33,69]. Most importantly, it avoids reaching a mass transfer equilibrium between the hydrogel-drug delivery system and the release media, which would slow down the drug release and would not be representative of the physiological situation where the drugs are absorbed/cleared within the surrounding tissues as soon as it is released [27].

#### 3.1.4. pH

The pH influences drug solubility [126], electrostatic properties [127], and interactions of the drug and the hydrogel polymer [128], and consequently can dramatically affect release profiles in some cases [61]. However, only a small amount of studies control pH in their release experiments. While pH may not impact all hydrogel drug delivery systems, controlling the pH at a physiologically relevant value is desired to best predict in vivo release. Zhao et al. first studied apigenin release from gellan gum-based hydrogels at pH levels ranging from 1–8 because the hydrogel drug delivery system was destined to the oral administration route, which is characterized by an acidic gastric pH and more neutral intestinal pH. The authors found that, at pH 4 and below, the drug release was sustained and achieved only 60% release within 24 h. However, pH increase created a quicker release, which, at pH 8, turned into a burst release within 6–8 h. This shift in release profile was explained by the release mechanism being diffusive under acidic pH and erosion controlled from physiological pH [61]. The pH should be adapted to the destined delivery site and application of the hydrogel drug delivery system. Nazli et al. used buffers at different pH to simulate the gastric system (pH 1.2, HCl 0.1 M), the large intestine medium (pH 5.5), the small intestine (pH 6.8, 0.1 M Phosphate buffer), and the blood (pH 7.4, 0.1 M Phosphate buffer) [102]. They also simulated an oral absorption and gastrointestinal release of the drug from the drug delivery system in vitro: First a release in gastric pH for two hours, then it was followed by a release of the same drug delivery system at pH 6.8, representing the large intestine for the next three hours, and finally a three-hour release at pH 7.4. The release studies were comprehensive in terms of pH, but the release media could have been elaborated more, i.e., containing gastrointestinal enzymes for the pH 1.2 and 6.8 or using plasma for the release at pH 7.4 [102]. The dissolution apparatus (Figure 3) is the best manner to precisely control the experimental parameters, and the usage of USP-defined release media to thoroughly mimic the release in the gastrointestinal tract (GIT) ensures the best possible predictions of in vivo release [65]. For hydrogel drug delivery systems that are to be implanted in areas other than the intestinal or gastric environment, the physiological pH of the study in most healthy areas is 7.4. The acidic cancer microenvironment was also considered and investigated for smart hydrogels with minimized drug release at physiological pH, whereas at acidic pH, the release was accelerated [13,129], enabling pH-sensitive, smart, and controlled release of anticancer agent. In the case of a wound, the pH also varies from slightly acidic (pH 7) for a simple cut and pH 4–5 during the healing process [130]. Upon bacterial infection, the pH can turn extremely acidic or slightly alkaline, depending on the bacteria species present [130]. Consequently, smart hydrogel drug delivery systems were developed to have a minimal release at physiological pH and an accelerated release at acidic pH and wound healing pH [131,132].

#### 3.1.5. Composition of the Release Media

Finally, the release media composition is decisive in creating experimental conditions that are physiologically relevant to the target microenvironment, providing the best prediction of in vivo release. Most release studies use PBS at pH 7.4 as it provides physiological pH and osmolality [7,27,59,60,62,63]. The ionic strength of the release media affects the drug solubility and release profiles. Several molarities of PBS buffer were tested for the release of verapamil hydrochloride from hydrophilic matrix tablets (based on Eudragit^®^ and Carbopol^®^) (Figure 2C), and the resulting release profiles showed a more controlled and sustained release for 0.05 M that reached around 50% after 24 h, whereas 0.1 M and 0.2 M provided a faster release that reached around 80% within 24 h. The higher ionic strengths were thought to impair the polymer’s molecular structure by increasing repulsive electrostatic interactions between charged polymer molecules. In addition, the ionic drug verapamil is more soluble at higher drug molarities. Both events, therefore, led to a faster release at higher drug molarities [113]. Other studies have reported the usage of simulated gastric fluid (SGF) and simulated intestinal fluid (SIF) since the focus of the drug delivery systems in question was to release drugs via the oral route [60,67]. For instance, Hu et al. developed a self-assembled polyelectrolyte complex hydrogel from salecan and chitosan for sustained release of vitamin C. Since the vitamin C molecules were destined for the oral route of administration, they used a more complex release medium as an in vitro model: They used SGF and SIF [12]. SGF contains water, pepsin, hydrochloric acid, and sodium chloride and has a pH of 1.2 [133]. SIF has a pH of 6.8 and contains water, potassium dihydrogen phosphate, sodium hydroxide, and pancreatin [134]. The release assay, which results are presented in Figure 2B, was performed in 50 mL of release medium at 50 rpm agitation, and 1 mL of the solution was sampled for analysis and refreshed for each time point. The approach of using a more complex medium than just PBS, and a more relevant media, which not only provides a suitable pH but also contains enzymes, proved to be successful in predicting the in vivo release of vitamin C. In their work, Hu et al. demonstrated the potential of using appropriate and highly physiologically relevant release media since the in vitro release profile correctly predicted the in vivo release [12].

While PBS provides physiological pH and ion osmolality, it does not contain any biological elements, such as proteins, antibodies, or cells, that would encounter the hydrogel-drug delivery system once implanted. To improve the in vitro release protocol and further mimic the situation occurring in vivo, more complex release media could be used. For example, the human serum contains all blood proteins, particularly enzymes, and cells, while also providing a physiological pH and ion osmolality. Several studies showed the potential of utilizing plasma to increase drug bioactivity [135] and impact the drug release kinetics [136] of hydrogel delivery systems. While it is acknowledged that release media containing biological elements such as plasma proteins present additional challenges of composition variability and quicker degradation compared to PBS, which is inexpensive and has a simple and standardized composition, the potential benefits of such sophisticated media to better predict in vivo release should be considered.

The impact of enzymes was explored for the release of the anticancer drug bufalin from an esterase-sensitive polymer made of oligo(ethylene glycol) monomethyl ether methacrylate and 3-((2-(methacryloyloxy)ethyl) thio)propanoic acid. For this polymer and any other compound that can be cleaved and eroded by enzymes, the in vitro release should integrate the specific enzyme to provide the most accurate release prediction. The authors compared the release with and without 10 units of esterase in PBS buffer at pH 7.4 and at 37 °C (Figure 2D). Unsurprisingly they found that the release was accelerated in the presence of the enzyme and reached beyond 80% in 40 h, while release in PBS alone was around 25% after 40 h [37].

### 3.2. Release Kinetics and Mathematical Modeling

Release kinetics curves can commonly present different phases from a linear increase characteristic of a constant amount of drug released over time (zero-order kinetics) [114], a constant percentage of the drug cargo released over time (first-order kinetics) [114], or a sudden first burst phase of release followed by a second more stable release until reaching a plateau [94].

Since cumulative drug release is complex and can be the result of a conjunction of several phenomena, e.g., diffusion, swelling, and others, it is common practice to investigate the mechanistic of release kinetics using different mathematical models. Mathematical models are beneficial to characterize and further explain release mechanisms. Although the fitting of release data is often used in drug delivery-related research publications, it is still not common practice. Caccavo et al. reviewed the trend in mathematical models used in the field of hydrogel-based drug delivery system and revealed that the main fitting model equations used are:

The zero-order kinetics Equation (2):

(2) $$\frac{M_{t}}{M_{\infty }}=kt$$

The first-order kinetics Equation (3):

(3) $$\ln\left(\frac{M_{t}}{M_{\infty }}\right)=kt$$

The Peppas or Korsmeyer–Peppas kinetics Equation (4):

(4) $$\frac{M_{t}}{M_{\infty }}=kt^{n}$$

The Higuchi Equation (5):

(5) $$\frac{M_{t}}{M_{\infty }}=kt^{1/2}$$

The Hixon–Crowell Equation (6):

(6) $$W_{0}^{1/3}-\;W_{t}^{1/3}=kt$$

Where *M_t_* is the total amount of drug released at a time *t*, *M_∞_* is the total amount of drug cargo to be released, *n* is the diffusivity exponent, *W_0_* is the initial amount of drug in the drug delivery system, *W_t_* is the remaining amount of drug in the drug delivery system at time t, and *k* is the kinetics constant [114,137].

**Figure 4:**
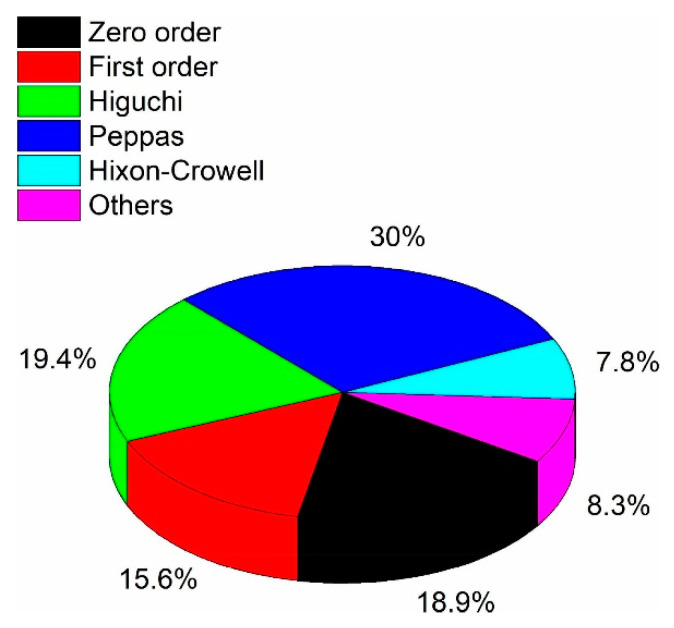
Relative use of mathematical equations used to describe in vitro drug release: Zero-order, first-order, Higuchi, Peppas, Hixon–Crowell equations and others, reported from research publications found when searching “modeling drug release from hydrogels” from 1980 to 2018. Reproduced with permission from [114]. Copyright Elsevier, 2019.

Figure 4 shows the relative use of mathematical models for hydrogel-drug delivery system release. The zero-order model corresponds to a constant release, where the same amount of drug is released per unit of time [137]. Conversely, the first-order model characterizes a release that is dependent on the drug concentration, where the same percentage of drug cargo is released per unit of time. It is often the case of sustained-release and matrix diffusion-controlled release [137]. The Higuchi model was initially designed to model drug release kinetics from ointments, films, and planar systems applied on the skin but have since been extended to three-dimensional dosage forms where the drug release is diffusion-driven only. As such, the Higuchi model is a particular case of the Peppas model (described in the next paragraph) and should not be used when the release is due to hydrogel swelling [114,138]. The Hixson–Crowell should be used to describe drug release from dosage forms that have changes in their diameter and surface area [137]. Consequently, it is not well-suited to model the drug release for hydrogel drug delivery systems [114].

The most frequently used model for hydrogel drug delivery release data is the Peppas equation [114]. This Equation (3) is highly informative because it indicates the type of release mechanism involved. In the case of a cylinder-shaped hydrogel-drug delivery system, if *n* < 0.45, the release mechanism follows a quasi-Fickian diffusion, and if *n* = 0.45, it follows Fickian diffusion. This means that the drug can diffuse through the hydrogel mesh because it is either much smaller than the mesh size and diffuses quickly. Alternatively, if the drug has a similar size to the mesh size, its diffusion is slower. If *n* > 0.89, it is referred to as Case II transport (zero-order kinetics), and the release is due to the swelling of the system. If 0.45 < *n* < 0.89, representative of anomalous transport, the release mechanism is a combination of diffusion and swelling. In the case of release by swelling, drug size is larger than mesh size; hence the drug is entrapped in the hydrogel and can only be released by deformation or degradation of the latter [4,139,140,141,142].

When the Peppas model indicates that the release is the result of an “anomalous transport” (0.43 < *n* < 0.89), diffusion exponent *n* values closer to 0.43 indicate that the release is closer to a diffusion-based release, whereas *n* values closer to 0.89 suggest that the swelling mechanism dominates [64,65,102]. On the other hand, values around 1 corresponded to a case II characteristic of a zero-order kinetic, and release is usually swelling or erosion controlled [64]. In a study by Zhao et al., the kinetic constant *k* and the diffusion exponent *n* of the Peppas equation were plotted against the pH to characterize the release of apigenin between pH 1 and pH 8. The obtained curves of *k* versus pH had an inflection point, indicating a switch in the release kinetics mechanism at pH 4 [61]. The Peppas diffusion exponent *n* was used to decipher the release mechanism, while the kinetics constant *k* was indicative of the reaction rate [61]. When the release mechanism is categorized as anomalous, the nonlinear Kopcha model can define the relative contribution of diffusion and relaxation to the drug release (Equation (7)).

(7) $$Q_{t}=At^{\frac{1}{2}}+Bt$$

Here *A* is the diffusional exponent, *B* the erosional constant, and *Q_t_* is the amount of drug released in time *t* [13,143].

In both their studies, Zhang et al. evaluated the release of BSA and 5-fluorouracil using the Peppas mathematical model, which successfully fitted the release data with *R*^2^ above 0.99. Cumulative release curves presented a first burst phase lasting for approximately the first 24 h, then a slower release phase, which eventually plateaued after 140 h. The diffusivity exponent *n* was above 0.45 for the cylindrical drug delivery system, delivering the model protein drug, which indicated that the release of BSA was a combination of diffusion and polymer chain relaxation (swelling) mechanisms. On the other hand, for the small chemical drug 5-fluorouracil, *n* values were below 0.45, indicating a diffusion-based release mechanism [58,118]. The obtained results were expected since the small chemical molecules are more prone to fast diffusion through the polymer matrix, whereas large protein molecules may be slowed down or retained by the mesh size, which would only be released by polymer deformation. This explanation is occulting possible electrostatic, hydrophobic, or chemical interaction between the drugs and the hydrogel. Furthermore, the authors calculated the diffusion coefficients *D* from both drugs and hydrogel formulations using the slope of the fitting curve of log-log plots of M*_t_*/M_∞_ vs. *t* [58,118]. The results showed that the more xanthan gum was present in the formulation, the lower the *D* value was for both drugs, implying that the higher content created more steric hindrance that decreased the diffusivity of the drugs.

In general, researchers use the zero-order, first-order, and the Peppas models to describe their release kinetics. However, for more complex release kinetics cases, the advised practice is to fit the release data into several models and see which model best fits the data, with typically higher R².

### 3.3. Statistical Analysis

Statistical analysis is an essential aspect of the scientific reporting of results, although often overlooked or incorrectly performed. The reviewed cumulative release studies often are presented using the mean and standard deviation, but in some studies, no statistical analysis was performed, limiting the relevance of results [60,61,64,102]. At best, the authors performed a one-way analysis of variance between all groups on the last day of release [6,43]. In their work, Lait et al. used the Student’s t-test to evaluate the significance of differences between experimental groups at the end of the release and at each time point [144]. In another study on siRNA release, the authors assessed statistical significance using repeated-measures ANOVA (Bonferroni–Dunn test) on days 7, 14, 28, and 35 [145]. These evaluations were not sufficient to identify significant differences between groups because they did not consider the ‘time’ variable. However, time is the most relevant variable in drug release as it will determine if the release kinetics, which can change over time. Time is an essential parameter in the statistical analysis of release profiles; otherwise, the analysis is incomplete. Various statistical models and software are available to perform a more comprehensive and complete evaluation of release curves. In our previous work, we evaluated the ‘time,’ ‘dose,’ and ‘GelMA hydrogel concentration’ parameters on the in vitro release of Abraxane^®^ [94]. We used a general linear model that provided a multivariate linear regression and fully integrated the time variable, and compared the GelMA concentration and drug dose over time, enabling us to make meaningful conclusions about the results. One software that should be mentioned is the MODDE Design of Experiments software. For instance, Leung et al. performed in vitro release studies in triplicate and presented the data as means ± one standard deviation. Interestingly, they used a Box–Behnken Design method to evaluate the impact of polymer composition on the gelation temperature and the release rate of methylene blue. This multiparametric statistical analysis was performed by the MODDE software, ensuring adequate model fitting and allowed a statistical prediction on the output parameter, e.g., the release rate, according to polymer compositions [59].

Overall, improvement should be made with broader integration of statistical analysis that takes the full curves into account in order to truly compare drug delivery systems. Statistical tools such as the MODDE software or a general linear model analysis using standard statistical software, advised as they provide a more reliable and comprehensive analysis.

## 4. Drug Diffusion Evaluation Methods

The diffusivity of drugs through the hydrogel matrix will significantly impact release rates and kinetics. Drug diffusivity is primarily dependent on the [mesh size/drug size] ratio, which controls steric interactions [4,146]. Thus, it is critical to incorporate knowledge of diffusive behavior when designing a hydrogel drug delivery system to obtain a release adapted to the desired application. However, information on therapeutic proteins and molecules’ diffusivity is not always available because assays are usually performed using inexpensive “model” molecules or protein instead of the more expensive drugs. Therefore, the diffusivity characterization frequently remains limited to model molecules such as BSA or dyes [41].

Cumulative release experimental data fitted with mathematical models, e.g., the Korsmeyer–Peppas model, are commonly used to inform about release mechanisms. Such models typically provide a computational determination of the drug partition coefficient *K* that can be defined as the concentration of the drug in the hydrogel polymer divided by the concentration of the drug in the free water contained in the hydrogel [147], the drug diffusion coefficient *D* (m^2^ s^−1^) that expresses the drug diffusion speed, the diffusion exponent *n*, which indicates the diffusion mechanism, the diffusion kinetics constant *k* characteristic of the diffusion rate. Nevertheless, the release assays take time and require large amounts of often expensive drugs.

The Franz cell diffusion assay is a state-of-the-art technique to evaluate drug diffusivity through skin and membranes. It can be used for and applied to two-dimensional hydrogel membranes.

Other experimental methods that are more direct and can monitor drug diffusion through three-dimensional hydrogel systems at a molecular scale provide information on drug diffusion complementary to the computational predictions. Such experimental methods are based on fluorescence imaging [39,40] and NRM spectroscopy [38]. Finally, the microfluidics approach [41] provides high adaptability and high throughput for drug diffusion studies of hydrogel drug delivery systems.

### 4.1. Computational Predictions

Fick’s second law can be directly applied to obtain the drug partition coefficient *K* and drug diffusion coefficient *D* in the case of one-dimensional drug diffusion that is characteristic of flat hydrogel drug delivery systems [27,62].

Regarding three-dimensional hydrogel drug delivery systems, the Korsmeyer–Peppas model has been the most used and provides the most information on drug diffusion prediction [114]. The model, which is derived from the second power law and was used in several studies to extrapolate the diffusion of solvents through hydrogels, has been well described since the late 1980s [148,149,150,151]. Singh et al. used the Korsmeyer–Peppas mathematical modeling to analyze a meropenem antibiotic release curve and extrapolate the diffusivity exponent *n*, the kinetics constant *k*, and diffusion coefficient D of the drugs through a poly(2-hydroxyethyl methacrylate) moringa gum hydrogel [152]. The previously mentioned parameters can be used to characterize different hydrogel formulations and crosslinking methods [60], thus, potentially aid the optimization phase of hydrogel drug delivery systems.

Zhang et al. carried out a study investigating the release of BSA (66.5 kDa) and 5-fluorouracil (130 Da) from xanthan-based hydrogels. As a reference, the values obtained for *D* values were in the range from 1.703 to 3.638 × 10^−11^ m^2^/s for BSA and from 2.012 to 6.671 × 10^−12^ m^2^/s for 5-Fu; and *k* values were ranging from 3.58 to 9.27 × 10^−3^ for BSA and from 1.888 to 3.963 × 10^−3^ for 5-fluorouracil [58]. They proposed a solution of Fick’s second law of diffusion, considering that their cylindrical hydrogel-drug delivery system sample was in perfect sink conditions, all surfaces were exposed to the release media (PBS). To compare the diffusion coefficient values obtained by the Peppas mathematical model, they used the Stokes–Einstein (Equation (8)) [58], which has been used to describe the diffusivity of a drug through the hydrogel matrix:

(8) $$R_{h}=\frac{K_{B}T}{6\pi \eta D}$$

where *R_h_* is the drug hydrodynamic radius, *K_B_* is the Boltzman constant, *T* is the absolute temperature, and *η* is the dynamic viscosity of the solvent (PBS, taken as 8.937 × 10^−4^ Pa·s). The hydrodynamic radius of BSA and 5-fluorouracil were measured using dynamic light scattering (DLS) in PBS at room temperature and found *D* values higher than those calculated by fitting the release data into the Peppas model. This was to be expected since the viscosity of PBS was used to solve the Stokes–Einstein equation, whereas the Korsmeyer–Peppas model fitted the release data that were impacted by the drug-polymer interaction. Thus the values obtained from the Korsmeyer–Peppas model were closer to reality [58]. These studies provided a comprehensive overview of how to calculate drug diffusivity via mathematical modeling and that is critical to consider the drug parameter, e.g., hydrodynamic radius, and the polymer parameters, e.g., viscosity.

Computational predictions help to characterize and optimize hydrogel drug delivery systems, yet they do not always fully reflect reality. A more elaborated and comprehensive experimental protocol is needed to resolve the Stokes–Einstein equation experimentally. Besides, it would be beneficial to compare the experimental data used in the Stokes–Einstein equation with the Peppas modeling of the release data to show whether the experimental and theoretical outcomes correlate.

Overall, the evaluation of the drug diffusion coefficient through a three-dimensional hydrogel through mathematical modeling does not easily predict the diffusion. The nature of the drug, e.g., hydrophobic drugs, large protein drugs, adds a layer of complexity since the drug diffusion out from the hydrogel drug delivery system can be altered by physical or electrostatic interactions.

### 4.2. Franz Cell Diffusion Assay

The Franz cell diffusion assay is designed to study the ex vivo transdermal diffusion of the drug from ointments. As such, it has been the gold standard method to study the topical drug permeation of skin [153,154,155]. The technique could also be applied to synthetic membranes and hydrogel drug delivery systems that are two-dimensional like membranes for transdermal drug administration [156,157]. The device is composed of an upper donor chamber where the drug-loaded hydrogel is placed and a lower acceptor chamber filled with a solution that is agitated via a magnetic stirrer and comporting a sampling port. The two chambers are separated by the membrane or skin that is the object of the study [158]. It can be used to assess both drug diffusion but also drug release. In this respect, the degree of agitation temperature, release, and sampling volume, release media pH, and composition are all inter-playing parameters [158]. The Franz cell diffusion assay was used to characterize drug diffusion in the case of an anti-protozoal drug and anesthetics for topical administration [159,160].

Potential limitations of this approach are the requirement of several Franz cell devices to provide sufficient statistical reliability of results and that the diffusion would be one-dimensional only. Consequently, this technique is mainly relevant for application where hydrogels are coating a substrate.

The following drug diffusion evaluation methods developed thereafter are focusing on three-dimensional hydrogel drug delivery systems.

### 4.3. Nuclear Magnetic Resonance Spectroscopy

Nuclear magnetic resonance (NMR) spectroscopy is a non-invasive technique used particularly for soft materials such as hydrogels. The method investigates the translational molecular motion during drug diffusion to effectively provide the drug coefficient D in m^2^/s for hydrogels [38]. The technique is based on the application of magnetic pulsed-field gradients that encode and decode the molecular mean-square displacement along the gradient direction [70]. The diffusion of ibuprofen, a well-known anti-inflammatory drug, through a 2,2,6,6-tetramethylpiperidine 1-oxyl-oxidized and cellulose nanofiber hydrogel, was measured by high-resolution magic angle spinning (HR-MAS) using the pulsed field gradient spin-echo (PGSE) NMR approach. The authors measured the diffusion coefficient of both ibuprofen and β-cyclodextrin-Ibuprofen and confirmed a 2-fold reduction of the drug diffusion coefficient when it was coupled with β-cyclodextrin [161]. The PGSE-NMR method was already discussed for Ibuprofen loaded in anionic agarose-carbomer hydrogel by Castiglione et al. [70] and described by others [162,163] that found diffusion coefficients in the same order of magnitude (× 10^−10^ m^2^/s to × 10^−11^ m^2^/s). Santoro et al. went further when they compared the theoretical self-diffusion coefficient of the small molecule sodium fluorescein with the diffusion coefficient experimentally obtained with HRMAS-NMR with PGSE. The theoretical coefficients were 1.4 × 10^−9^ ± 0.07 m^2^/s and 1.2 × 10^−9^ ± 0.06 m^2^/s, in two different hydrogel formulations made of agarose and carbomer 974P, whereas the NMR coefficients were, respectively, 1.32 × 10^−9^ ± 0.13 m^2^/s and 1.30 × 10^−9^ ± 0.13 m^2^/s, which were very close from the calculated values, yet the replicate numbers of the experimental groups were not reported nor any statistical test to validate equivalence performed. Nonetheless, this work hinted at the validity of the HRMAS diffusion-ordered spectroscopy NMR technique to accurately and quickly estimate the diffusion coefficient for drugs of small steric hindrance, which could not be obtained elsewhere [164]. However, the approach nevertheless seemed most suited to small molecules and was found to be limited to molecular weight below 20 kDa [165].

The potential of HRMAS-NMR is excellent to obtain direct, very accurate, robust, and precise information into the molecular mechanistic of drug diffusion. Additionally, it has the advantage of providing this information for non-fluorescent molecules. Nevertheless, it remains costly and, what is more, the method seems limited to small and fast diffusing ions or molecules that present no steric hindrance through the hydrogel, e.g., ibuprofen, sodium fluorescein, ions [166,167] and is not well suited to macromolecules that are slow diffusive, i.e., above 20 kDa [165].

### 4.4. Fluorescence Microscopy Techniques

#### 4.4.1. Fluorescence Recovery after Photobleaching

**Figure 5:**
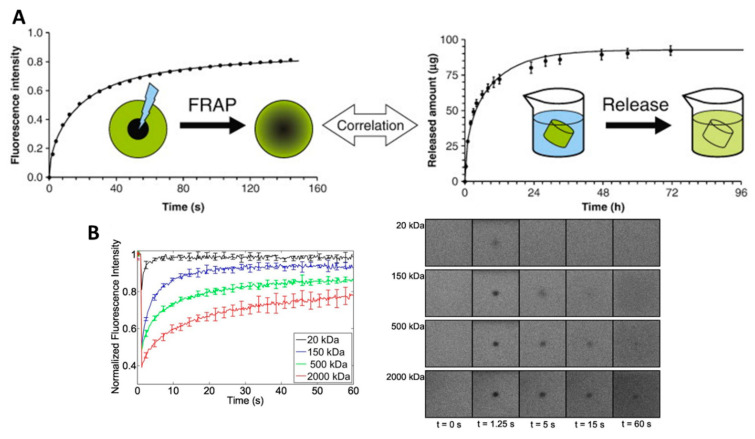
Fluorescence recovery after photobleaching (FRAP) measurements correlates to the in vitro drug release. (**A**) FRAP measurements help determine the drug diffusion coefficient in the hydrogel drug delivery system and predict drug release profiles. Graphical abstract reproduced with permission from [165]. Copyright Elsevier, 2010. (**B**) Normalized fluorescence recovery curves of different sized FITC-dextran molecules (20, 150, 500, and 2000 kDa) in hydrazine crosslinked hydrogels and the corresponding images of the zone of interest from 0 to 60 s. Data are shown as means ± standard deviation, *n* = 5. Reproduced with permission from [89]. Copyright Elsevier, 2019.

Fluorescence recovery after photobleaching (FRAP) can be defined as a microscopy-based technique that is used to experimentally evaluate the diffusion coefficient of molecules through tissues or soft materials (Figure 5). FRAP has been used in the hydrogel-drug delivery field to evaluate the diffusion kinetics of molecules, and most often proteins [39,40] or dextran molecules of similar size to protein and growth factors, i.e., ranging from 20 kDa to 2000 kDa [89,165,168,169,170,171], through hydrogels. While small dye molecules frequently possess autofluorescence properties, large molecules, e.g., proteins, need to be labeled with a fluorescent label, often FITC, before being encapsulated in the hydrogel. In principle, in a FRAP assay, a region of interest of the drug delivery system undergoes photobleaching. Then, the kinetics of fluorescence intensity recovery in the region of interest are measured (Figure 5B right panel). A fit to the recovery curve yields the diffusion coefficient (Figure 5B left panel). Typically, FRAP is used to correlate the diffusion coefficient of molecules throughout the hydrogel drug delivery system with the mesh size of that same hydrogel system [89]. More precisely, for a fixed hydrogel mesh size, the bigger the molecule, the slower its diffusion [89]. Karvinen et al. proposed an experimental setting to evaluate the diffusion rates of different size FITC-labeled dextran molecules through a hydrogel using FRAP [89]. Their FRAP data was congruent with their rheology experiments regarding mesh size and molecule diffusion properties. They demonstrated that the storage modulus increased with a decrease in mesh size and increased cross-linking density. Similarly, the diffusivity of the FITC-dextran particles, shown in Figure 5B, decreased from 80 ± 4 μm^2^/s to 0.05 ± 0.03 μm^2^/s with an increase in dextran molecular weight from 20 kDa to 2000 kDa. The authors were also able to extrapolate their FRAP experimental data into a virtual simulation diffusion through different hydrogels that could be applied to cells [89].

Conversely, at a fixed molecule size, the diffusion coefficient decreases with mesh size. This property was demonstrated by Kaemmerer et al. using FITC-dextran (70 kDa) and studying its diffusion coefficient in 2.5%, 5%, and 7% GelMA hydrogels [168]. It was also shown by Henke et al. for poly(ethylene glycol) (PEG)-based hydrogels at different molecular weights containing different sizes of FITC-dextran molecules [170], and also confirmed for FITC-labelled BSA, Lysozyme, and IgG proteins in poly sulfobetaine methacrylate hydrogels of different mesh sizes [39].

A very instructive study was performed by Brandi et al. [165], where they compared and correlated the diffusion coefficient of FITC-dextran (20 kDa, 150 kDa, and 2000 kDa) in 5% and 10% poly(ethylene glycol) (PEG) hydrogels, obtained by mechanical testing and swelling studies, FRAP, pulsed field gradient NMR spectroscopy, and the cumulative release profiles (Figure 5A). They used as references the calculated values obtained from their swelling and mechanical studies. Overall, the diffusion coefficients calculated from the FRAP experiments were logically decreasing from 5% PEG to 10% PEG and as the FITC-dextran size increased. In addition, the diffusion coefficients measured by FRAP were matching the predicted coefficients for 2000 kDa and 150 kDa FITC-dextran. However, the 20 kDa FITC-dextran, which was expected to diffuse fast (at 71.1 µm^2^/s and 67.3 µm^2^/s for PEG 5% and PEG 10%), actually displayed a slower diffusion during the FRAP measurement (20.2 ± 0.2 µm^2^/s and 18.1 ± 0.2 μm^2^/s, for PEG 5% and PEG 10%). It is to be noted that the NMR measurement of the diffusion coefficient was limited to 20 kDa FITC-dextran only. Finally, the diffusion coefficients obtained from the cumulative release data were higher than those from the FRAP experiment because the two experimental approaches did not measure the same event. The release experiments resulted from a passive diffusion based on gradient concentration, whereas the FRAP technique measures the self-diffusion of molecules that do not rely on gradient diffusion [165].

In summary, the FRAP technique is demonstrated to be suitable for slow diffusive molecules, whereas the pulsed field gradient NMR spectroscopy is ideal for fast-diffusive molecules. Altogether, the FRAP and NMR spectroscopy can be used as complementary tools to the mathematical calculation of the predicted diffusion coefficient and the cumulative release providing a diffusion coefficient based on gradient concentration diffusion.

Although the FRAP technique is well established in the hydrogel drug delivery field to measure the diffusion of fluorescent molecules, one important limitation is that the process of photobleaching impacts the fluorescent labels only, and thus does not mean that the therapeutic molecules or protein are actually physically released from the zone of interest. This means that the unbleached fluorescent molecules, initially outside of the bleached zone, when diffusing into the zone of interest, can be slowed down by already present molecules that were photobleached. Thus, it leads to underestimating the real diffusion coefficient. Moreover, the experimental set-up, e.g., the photobleaching parameters, strongly affects the capacity of measurements; therefore, there is a risk of artifacts [172]. As such, a standard procedure needs to be determined for each system by systemically correlating with mathematical and actual data.

#### 4.4.2. Fluorescence Correlation Spectroscopy

Another technique that has been used alongside FRAP for the diffusion of protein [173] and living cells [172,174,175] is Fluorescence Correlation Spectroscopy (FCS). Similar to FRAP, it is used to measure the diffusion coefficient of fluorescent molecules through soft tissues, but it does not use photobleaching. Instead, it follows the movements of fluorescent molecules in a three-dimensional volume, which is spatially resolved through optics. The light is focused on the sample and measures fluorescence intensity fluctuations [175]. As is the case with FRAP, the FCS technique enables the determination of the diffusion coefficient. Unlike FRAP, FCS cannot measure the partition coefficient but can provide molecule concentration without calibration [176]. With the FSC technique, the beam intensity should be sufficient to measure the fluctuations of fluorescence intensity against the noise but not too intense to minimize photobleaching [177].

FCS was used to measure the diffusion and interaction of model proteins in crosslinked PEG systems and compared the obtained results with the estimated effective diffusion coefficients calculated from a cumulated release curve [178]. While both methods measured the diffusion coefficient from different scales, i.e., molecular and single-molecule scale for FCS and macromolecular scale for the bulk release assay, the yielded diffusion coefficients were consistent in terms of values and also showed the same trends. The FCS provided additional information on the hydrogel-protein interactions occurring during the crosslinking and causing steric hindrance and diffusion impairment [178]. FCS was also used to measure the diffusivity of a fluorescent immunoglobulin G in a poly(N-isopropylacrylamide) thermoresponsive hydrogel [179], the diffusivity properties of a polyacrylamide hydrogel using different size of dextran molecules [180,181], and the diffusivity and dynamics of BSA in a pluronic F127 hydrogel [182].

A study focused on comparing the FRAP and FCS methods in simultaneous measurement of protein diffusion in a lipid membrane [175]. Their findings suggested that the intense photobleaching of FRAP did not impact the solute diffusion; therefore, both FRAP and FCS yielded similar changes and coefficient values. However, the fitting and bleach corrections are more accurate for FCS compared to FRAP; thus, they can bring uncertainties in the FRAP technique. On the contrary, FRAP has lower requirements on noise/signal ratios than FCS, which means a reduction of bleaching intensity and potential sample damage. Finally, FCS can do complete mappings of diffusion coefficients of particles for every pixel of an image with higher spatial and temporal resolution [175].

### 4.5. Microfluidics

Microfluidic platforms can be a versatile tool for testing the drug diffusion from hydrogels on a microscale and should be a critical part of developing a hydrogel-drug delivery system. The approach has the advantages of versatility because of the modularity of such platforms and because of increased throughput compared to other experimental methods.

**Figure 6:**
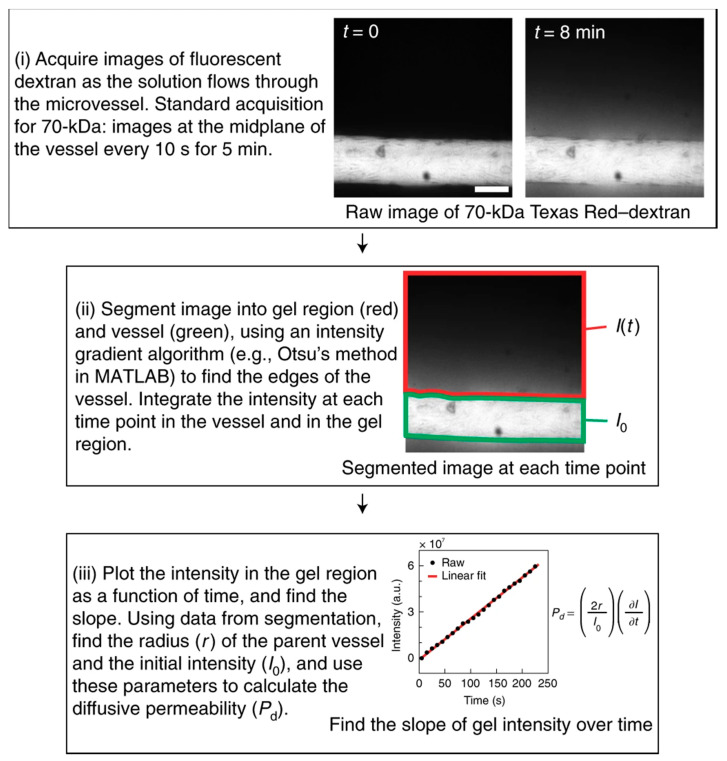
Quantifying the diffusive permeability of vessels in the human-engineered microvessel platform. (**i**) Images are taken in time-lapse to follow the flux of 70 kDa dextran. After 8 min, an image shows the diffusion of the dextran molecules into the collagen gel. The frequency and number of images necessary need to be optimized for each molecule and experimental condition. Scale bar = 70 µm. (**ii**) Next, the images are segmented into the gel region *I(t)* in red and the vessel region *I_0_* in green. *I(t)* is the gel intensity to be measured and compared to the initial intensity *I_0_* in the vessel. (**iii**) The intensity *I(t*) of dextran plotted as a function of time is used to determine *Pd*, the diffusive permeability of the vessel. Reproduced with permission from [183]. Copyright Springer Nature, 2019.

Polacheck et al. proposed a protocol to fabricate a human-engineered microvessel microfluidic platform [183]. The platform enables evaluating the permeability of the 3D extracellular matrix (ECM) tissue surrounding the microvessel (Figure 6) [183]. The hydrogel was injected in a mold and crosslinked around a needle to fabricate the platform, which was removed post-crosslinking, thereby creating a straight 3D lumen, with the diameter controllable through the size of the needle used. The authors successfully accommodated several hydrogel types, including gelatin hydrogels and collagen I-Matrigel ECM in the microfluidics platform. The permeability was assessed for 70 kDa Texas Red-dextran model molecules into a collagen hydrogel seeded with human umbilical vein endothelial cells by confocal imaging. The protocol allowed for a 5-minute fast permeability assay by monitoring the diffusion of fluorescent dextran molecules in the hydrogel when perfused into the lumen (Figure 6 (i)). Confocal images were taken every 10 s, and the fluctuation of fluorescence intensity in the lumen and surrounding hydrogel were used to determine permeability with MATLAB (code and method available in the supplementary data provided by of Polacheck et al. [183]) (Figure 6 (i) and (iii)). The concept of this platform can be used to study hydrogel permeability to different solutes, and it enables high-resolution confocal microscopy of the elements in the device. Indeed, the authors proposed a protocol for the rapid measurement and quantification of the hydrogel permeability to fluorescent molecules. A similar platform was designed by Shin et al. and used to study VEGF (40 kDa) diffusion gradient in collagen I hydrogels at different concentrations. It successfully combined experimental data and computational simulation to predict the diffusion coefficient at 5.8 × 10^−11^ m^2^/s [184]. Fluorescent-dextran diffusion coefficient was also investigated using microfluidic vascular cell culture platforms and fluorescence microscopy imaging, reaching congruent diffusion coefficient values of VEGF-sized dextran: 6.6 × 10^−11^ m^2^/s [185,186,187,188]. Another group elaborated a blood–brain barrier microfluidic platform and evaluated the diffusion coefficient of 4 kDa and 40 kDa of FITC-dextran molecules [189]. They found diffusion coefficients comparable to previous blood-brain barrier diffusion studies and in vivo measured diffusion coefficients, thus validating their microfluidic model.

**Figure 7:**
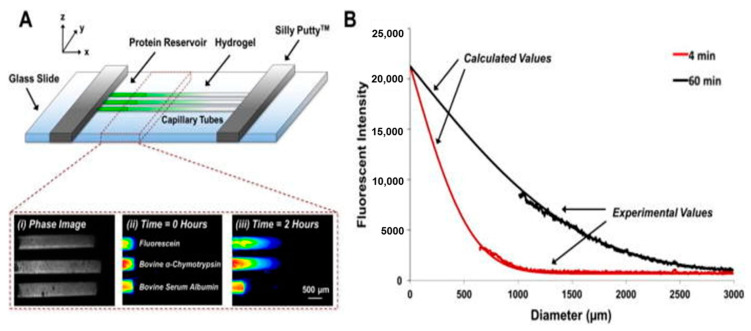
Diffusion in capillary tube experimental set-up. (**A**) Three capillary tubes were affixed to a glass slide (approximately 0.5 mm apart) using Silly Putty™ and filled with hydrogel and protein solution for microscope imaging. (**i**) Capillary tubes filled with 2% (*w*/*v*) collagen hydrogels bright field imaging. Fluorescence imaging of fluorescein-labeled bovine α-chymotrypsin and fluorescein-labeled bovine serum albumin diffusing through collagen hydrogels at (**ii**) 0 h and (**iii**) 2 h. (**B**) Fluorescence intensity experimental profiles were obtained from images at 4 and 60 min after the diffusion starts. Theoretical and calculated fluorescence profiles. Reproduced from [41] under a CC BY 4.0 license.

The concepts of the previously mentioned microfluidics 3D cell culture platforms were extracted and adapted by Hettiaratchi et al. (Figure 7) to run fast-measurements of protein diffusion coefficients through collagen, alginate, and PEG hydrogels. The diffusion of fluorescein-labeled model proteins (bovine α-chymotrypsin 25 kDa, bovine serum albumin 63 kDa, and human immunoglobulin G 150 kDa) was evaluated (Figure 7A), and the experimental data were used to create a computational prediction model for the diffusion of protein (Figure 7B). They demonstrated that diffusion coefficients decrease with increasing molecule size [41], and this relationship was also demonstrated elsewhere [190]. Hettiaratchi et al. went further and used their model to predict the diffusion coefficient of the protein of interest bone morphogenetic protein 2 (BMP-2) and compared it with the experimental data, which successfully confirmed the prediction. Therefore, the authors proposed a straightforward and robust capillary assay and mathematical model to measure and predict the diffusivity of therapeutic proteins through hydrogel-drug delivery systems [41]. This approach was used by Marshall et al. in a perfusion-flow bioreactor to develop a computational model to predict macromolecule diffusion in hydrogel-drug delivery systems using a 70 kDa FITC-dextran molecules as a model [191].

Finally, Evans et al. compared NMR with microfluidics effluent and optical techniques to measure the molecular diffusion coefficient of sulforhodamine 101, acid blue 22, and methylene blue dye through PEG-diacrylate (PEG-DA) hydrogels [192]. All three methods gave congruent diffusion coefficients within the same order of magnitude, with the NMR being the most precise, but the microfluidic channel platform enabled detecting an anomalous diffusion for acid blue 22, which was not observed with NMR. Altogether, the three methods combined provided adequate coverage and understanding of the molecular diffusion of small molecules through broadly used hydrogel drug delivery systems [192].

Microfluidic platforms are versatile, flexible, easy to use, and promising tools to study the molecular diffusivity and choose the appropriate material or drug when designing a hydrogel-drug delivery system. Nevertheless, the experimental set-up, e.g., the microscope, the objective, the concentration of fluorescent molecules injected, the flux used, the method of injection, frequency of imaging, can significantly affect the results and thus requires significant empirical optimization.

## 5. In Vitro and In Vivo Evaluation of Hydrogel Drug Delivery Systems

### 5.1. In Vitro

Despite being paramount to the understanding of the drug release kinetics and mechanisms, which are necessary to the design and development of hydrogel drug delivery systems, saline release assays are inherently limited in providing a range of efficacy against the target cells or tissue. Without in vitro testing, the relevance of the drug delivery system cannot be validated. Furthermore, in vitro assays provide insights on the required therapeutic dose range, which can then be translated in vivo [193]. Before preclinical/clinical application, the hydrogel drug delivery systems’ efficacy should be tested in vitro on relevant cell culture models. Historically drug effects have been primarily studied in two-dimensional (2D) cell cultures [194,195]. However, three-dimensional (3D) cell culture models are increasingly investigated as they provide a more physiological microenvironment for cell culture and drug testing [196,197].

#### 5.1.1. 2D In Vitro Models

2D cell cultures based on cells propagated in monolayers have been used as in vitro models to test mostly the anticancer efficacy [94], tissue and cell proliferation/differentiation [7,57,72], or antibacterial properties [198] of drug delivery systems.

First, hydrogels are tested for the cytocompatibility of the polymer. The primary method used is to encapsulate cells in the hydrogel drug delivery system and measure cell viability [10,72]. Then, hydrogels designed to deliver drugs are evaluated using relevant cell lines to test the drug’s anticancer or antibacterial activity, as well as tissue regeneration properties [7,57,72,94,198]. A hydrogel delivery system is usually compared with the same drug directly injected in the cell culture media (positive control) and an untreated group that serves as a negative control group [43,90]. After several days of drug treatment, the viability of cells are usually assessed via Alamarbue^®^ [7,94] or MTT [43] assays, which indicate the metabolic activity of cells. Alternatively, cell proliferation can be measured by DNA content (Picogreen assay) [72] or using a CCK-8 kit [13]. To visualize cell morphology, Rhodamine B and 4′,6-diamidino-2-phenylindole (DAPI) staining for cell cytoskeletal F-actin and nuclei, respectively, can be used [13]. Cell viability is usually directly assessed using live/dead staining (calcein and ethidium bromide to visualize live and dead cells, respectively) [13,72]. In the case of cancer cells and anticancer drugs, a higher caspase activity can also indicate the anticancer activity because the latter are involved in cellular apoptosis, which can be tested using detection caspase kits (e.g., Annexin V-FITC Apoptosis Detection Kit) [13].

The efficacy of hydrogel drug delivery systems delivering drugs to promote tissue regeneration requires additional tissue staining techniques and gene or protein analysis to evaluate tissue differentiation [57,72]. For example, chondrogenic differentiation of human adipose mesenchymal stem cells (hAMSCs) was assessed in 2D cell culture to test a kartogenin-loaded chitosan hydrogel for local tissue regeneration [72]. Reverse transcriptase qPCR results confirmed a higher expression for chondrogenic differentiation marker genes, i.e., *COL2A1*, *SOX9*, and *ACAN* for pure kartogenin and kartogenin-loaded hydrogel groups. Immunofluorescence analysis showed an increase in collagen type II and SOX9 protein production for kartogenin groups. Therein the immunofluorescence staining supported the PCR results by confirming enhanced chondrogenic differentiation [72].

The zone of inhibition assay has been the gold standard to evaluate the antibacterial efficacy of drugs and drug delivery systems. Briefly, the microorganism is homogeneously spread on an agar plate, and then the antibacterial devices are placed on the agar culture. After an incubation period at 37 °C, the growth inhibition zones are visible and measured [15]. It is a quick, inexpensive assay that provides a visual and quantitative measure of the antibacterial effect [199]. As a result, the method was used for several antibacterial hydrogel drug delivery systems [6,101,198].

While broadly used by the scientific community because of its inexpensive and easy usage, cell culture on 2D surfaces present the main limitation of physiological cell-ECM and cell-cell interactions [196]. Monolayer cell culture induces an unphysiological change in cell morphology from 3D as observed in the body to a flat, 2D morphology with forced polarity, causing cells to behave aberrantly and display unnatural drug responses [200]. When organized in a tissue, on the other hand, cells have dynamic interactions with adjoining cells and extracellular matrix environment allowing transmission of mechanical and biochemical stimuli regulating cellular behavior [201]. It is this 3D communication that is essential to tissue homeostasis and specificity [202], as well as cell cycle regulation, cell proliferation, differentiation, migration, or apoptosis [203].

#### 5.1.2. 3D In Vitro Models

3D cell culture models are mostly based on hydrogel or scaffold technologies [5]. They have shown superiority over 2D cell culture since they mimic and restore the cell–cell and cell-extracellular matrix dynamics of native tissue [204,205]. More precisely, 3D cell culture models’ benefits are a more natural extracellular matrix, more accurate physiology, cellular and molecular responses, increased cell longevity, functionality, growth and proliferation, a gene and protein expression closer to in vivo situations [200]. Consequently, they are investigated to decipher pathology mechanisms [206,207,208] for drug testing and screening [209,210,211] and predict the response of an organism on the cellular and sub-cellular level [197].

**Figure 8:**
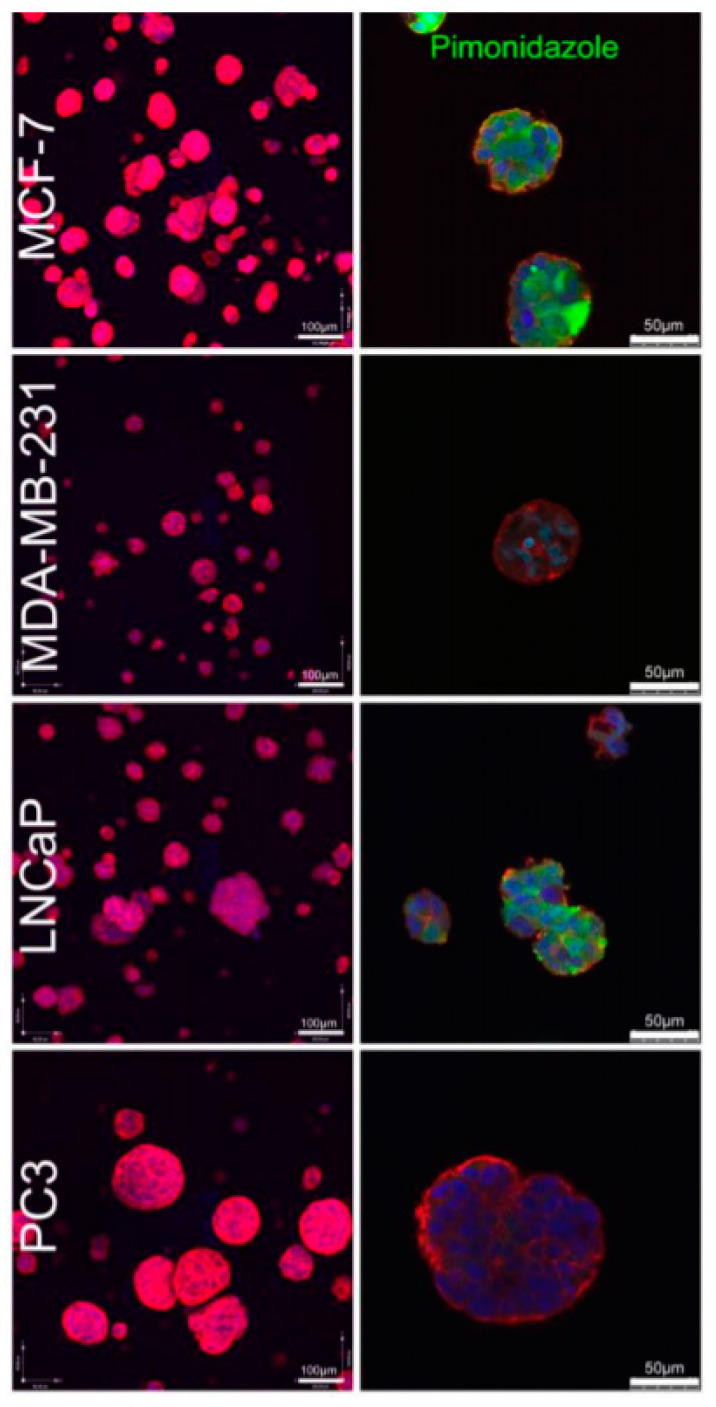
3D tumor formation. 3D confocal images of tumor microenvironment for MCF-7, MDA-MB-231, LNCaP, and PC3 cells cultured in starPEG-heparin hydrogels for 14 days. Cell morphology can be seen by Hoechst (blue) and phalloidin (red) immunostaining, while hypoxia can be visualized in green via pimonidazole staining. Scale bar = 100 μm for the left column and 50 μm for the right column. Reproduced with permission from [42]. Copyright Elsevier, 2015.

In our previous work, we have tested a range of Abraxane^®^ doses (from 0.9 µg/mL to 300 µg/mL) against breast cancer cell lines MCF-7 and MDA-MB-231 both in 2D and 3D cell culture configuration [94]. We showed that cellular metabolic activity was lower in 2D than in 3D configuration for both cell lines and all drug concentrations [94]. This confirmed that 3D cell culture models investigated in the study were more resistant to drugs than monolayer cell culture. The 3D model was more closely mimicking native tissue as cells grew to form aggregates or spheroids, allowing more physiological cell–cell contacts and signaling. All metabolic activity profile against dose were similar. Bray et al. also demonstrated the superior drug resistance of breast cancer MCF-7 and MDA-MB-231, and prostate cancer LNCap and PC3 cell lines cultured in 3D spheroids (Figure 8) and treated with epirubicin and paclitaxel [42].

Cells cultured in 3D were used to access the cytotoxicity of drugs released by various hydrogel drug delivery systems [7,57,72,90,93,94]. The hydrogel efficacy to co-deliver the drug resveratrol and a plasmid DNA coding for a vascular endothelial growth factor (VEGF), was assessed by seeding human umbilical vein endothelial cells (HUVEC) and the drugs in the same hydrogel drug delivery system. The hydrogel drug delivery systems were degraded at specific time points, and an ELISA assay was used to quantify VEGF expression. The hydrogels that contained the plasmid DNA induced a significantly higher VEGF expression, which also increased over time. Thus, they demonstrated potential as drug delivery systems for wound healing [10]. Osteogenic cell differentiation induced by the delivery of siRNA from a hydrogel was also assessed in a 3D cell culture configuration, where both the cells and siRNA were encapsulated in the same hydrogel drug delivery system, by Carthew et al. [93]. Finally, a 3D breast cancer cell in vitro model was used to demonstrate the cytotoxic efficacy of our GelMA hydrogel system, delivering Abraxane^®^ [94].

Despite the evident advantages of 3D cell culture models that established a more physiologically relevant system, they still present some limitations. The primary type of 3D cell culture model is based on utilizing hydrogel scaffolds to encapsulate cells [5]. Consequently, each model must be optimized to provide the targeted cell line’s best environment to be cultured to reach high cell viability [5]. While co-cultures provide a more accurate representation of the tumor’s in vivo biology, such a model also requires a great deal of optimization to combine the cell types with the right balance [42,212]. Finally, the hydrogel used to encapsulate the cells is a barrier limiting the drug penetration into the hydrogel. That limitation is characterized by the drug diffusion coefficient through the hydrogel or hydrogel permeability [213]. Moreover, the hydrogel stiffness and its permeability can evolve if the hydrogel is degradable by the enzymes produced by cells over time [214]. This entails that the diffusion of the drug through the hydrogel can also evolve.

An alternative to the antibacterial zone of inhibition bacterial susceptibility assay is to use liquid bacterial broth to test antibacterial drugs. Leung et al. evaluated the antibacterial properties of their hydrogel-DDS using bacterial culture broth to assess growth inhibition against three MRSA strains. The bacteria-killing efficiency was then measured in CFU/mL [59]. *S. aureus* (MRSA) and Gram-negative *E. coli* (ATCC 25922) were cultured in a broth to test the antimicrobial properties of a methacryloyl-substituted recombinant human tropoelastin (MeTro)/GelMA (MeTro/GelMA) composite hydrogel releasing an antibacterial peptide [215]. This antibacterial assay is derived from the microdilution assay for antimicrobial susceptibility. While the disc diffusion/zone of inhibitions assay is semi-quantitative, the broth microdilution is quantitative [199,216]. Both tests should be used in a complementary manner since they evaluate the microorganism response to antibacterial agents in 2D and in a liquid.

Tao et al. led a comprehensive assessment of the antibacterial efficacy of their antibiotic and anti-inflammatory hydrogel drug delivery system. They used 2D cell cultures of fibroblasts (NIH3T3) and human embryonic kidney 293 cells (HEK293) to assess the cytotoxicity of the hydrogel using CCK-8 and LIVE and DEAD staining assays to assess cell viabilities [6]. In parallel, since the application of their work was wound dressing, they evaluated the bacterial broth growth inhibition and zone of inhibition capabilities of their system against *E. coli*, *S. aureus*, and *P. aeruginosa*. Finally, they developed and used a 2D, in vitro, wound infection model by co-cultivation of HEK293 and NIH3T3 cells and *S. aureus* or *P. aeruginosa* on an LB agar plate [6]. They used a co-culture of different bacteria and mammalian cells to mimic the clinical context of their application closely. While the establishment of such a co-culture model must have required a great deal of optimization, it elegantly showed the effect the hydrogel drug delivery system has on both cell types when cultured together, and the interplay between both cell types in response to the antibiotic release.

3D in vitro cell culture models should more readily be investigated as they provide the best tissue response mimicry. The next generation of in vitro cancer models being developed and worthy of particular attention are the 3D co-culture of cancer cell lines [42]. Additionally, using patient-derived cells would provide more insight and personalized cellular response or susceptibility to drug treatments and guide therapeutic choice [217].

### 5.2. In Vivo

Before human clinical studies, preclinical animal models offered great insight into the response of the organism response to drug delivery systems. The purpose of in vivo studies, mainly performed on murine models, is to provide a complex biological platform to assess the efficacy of the drug release and the translatability of the drug delivery systems [218]. The goal of animal preclinical studies is to assess pharmacodynamics, the efficacy of the drug delivery device in terms of dose-ranging and toxicity, or to investigate the pharmacokinetics of the drug release, which refers to the absorption distribution, metabolization, and excretion of the drug [219,220,221,222]. As such, they are a critical milestone in the development of a drug delivery system. To this effect, in vivo models are becoming increasingly complex [223].

Hydrogel drug delivery systems can be used to deliver drugs using different administration routes, which makes them attractive biomaterials for that purpose [20]. In fact, hydrogel drug delivery systems can be applied via enteral/oral administration route [12,21], local implantation in the body (parenteral route) [22,23], but also the topical route, which is usually more relevant for two-dimensional hydrogel drug delivery systems [24,25,26].

Herein, the focus was put on in vivo studies investigating the efficacy of three-dimensional hydrogel drug delivery systems to be delivered via the oral administration route or locally implanted in the body (parenteral application).

**Figure 9:**
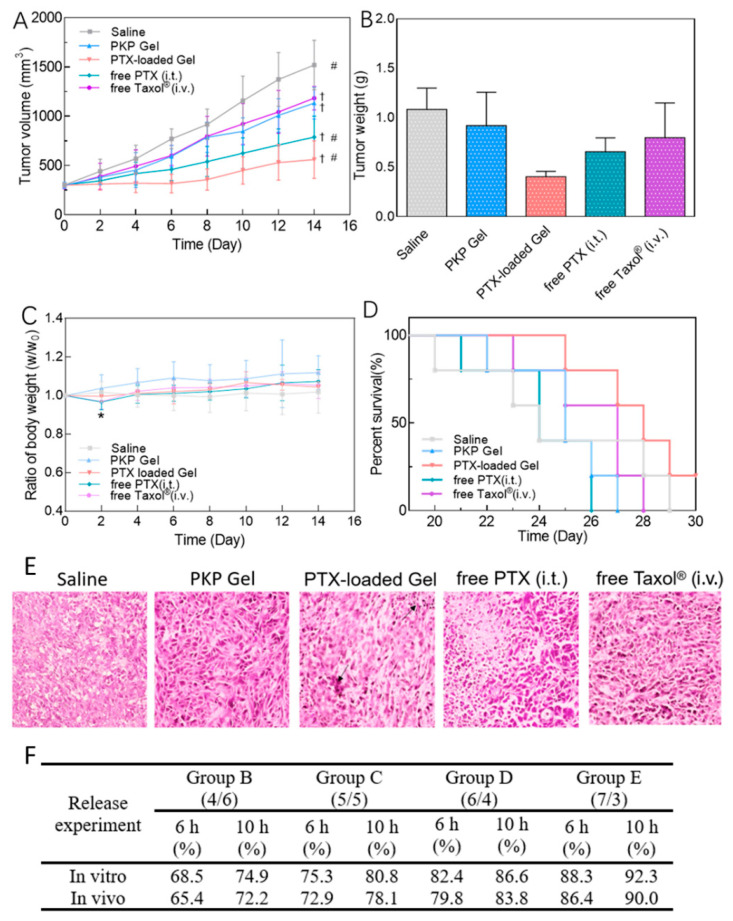
In vivo breast cancer model for tumor inhibition. Mice bearing 4T1 cells were treated with saline solution, polysaccharide hydrogel (PKP Gel), paclitaxel-loaded hydrogel (PTX-loaded Gel), free paclitaxel intra-tumoral injection (free PTX (i.t)), or free Taxol^®^ intra-veinous injection (free Taxol^®^ (i.v)). (**A**) Tumor volume (*n* = 9, means ± SD). (**B**) Tumor weight on day 14 (*n* = 3, means ± SD). (**C**) Ratio of body weight changes (n = 9, means ± SD). (**D**) Survival rate of mice until day 30 (*n* = 3). (**E**) Hematoxylin and eosin staining of tumor tissue after 14 days. Image obtained at 400× magnification with optical microscope and arrows indicate cell necrosis. Reproduced with permission from [43]. Copyright Elsevier, 2020. (**F**) Comparison of in vitro and in vivo amount of vitamin C from polyelectrolyte hydrogels (salecan/chitosan ratio = 7/3, 6/4, 5/5, and 4/6). Reproduced with permission from [12]. Copyright Elsevier, 2020.

In vivo study of hydrogel drug delivery systems generally evaluate drug release indirectly by assessing drug efficacy [15,43,44]. The main outputs for tumor inhibition models are the tumor volume and size, the weight of mice over time, and the survival rate of the animals. In parallel, histological stainings of tissues post-sacrifice complete the datasets. These tools were used by Jang et al., who tested their paclitaxel loaded-polysaccharide hydrogel drug delivery system in mice [43]. They monitored the tumor volume (Figure 9A), the tumor weight (Figure 9B), the bodyweight ratio (Figure 9C), the survival rate (Figure 9D), and performed histological stainings after animal sacrifice (Figure 9E).

The authors showed that all treated groups led to an inhibitory effect of the tumor except for the saline control group. The group of paclitaxel-loaded hydrogel exhibited the highest antitumor efficacy (Figure 9A,B). Hematoxylin and eosin (H&E) staining of the tumor post sacrifice confirmed higher levels of necrotic tissue for paclitaxel-loaded hydrogel, which indicated the superior efficacy of the hydrogel delivery system over free paclitaxel and taxol injections (Figure 9E) [43]. Similar in vivo studies were performed to assess the anti-tumor activity of paclitaxel released from gelatin hydrogels [224] and prospidine delivered by dextran phosphate-based hydrogels for local cancer therapy [44] using the animal survival rate and histology staining as common criteria.

In tissue regeneration applications, histological analysis is predominant in evaluating the efficacy of the drug delivery system [10]. For example, in vivo release of sinomenium from GelMA/Chitosan nanoparticles was assessed for osteoarthritis treatment of murine knee joint [225]. The Osteoarthritis Research Society International (OARSI) score, a semi-quantitative histopathological grading for cartilage degradation, was used to evaluate the resected tissues. Additionally, immunohistochemistry staining was used to detect the expression of cartilage degradation markers [225]. A similar rabbit in vivo study utilized histological (Hematoxylin/Eosin and Safranin-O staining), immunohistochemical (Anti-collagen II immunostaining) assays, and a qualitative grading of the osteoarthrosis process to assess the efficacy of Naproxen and Dexamethasone delivered by hydrogels for cartilage regeneration [7]. Another study tested a hydrophobically-modified gelatin hydrogel for growth factor delivery with an in vivo rat model. Briefly, they implanted the hydrogel samples subcutaneously with a control group treated with a saline injection instead of the hydrogel implantation, a group implanted with the drug-free hydrogel, and a third group implanted with a growth factor-loaded hydrogel. After sacrificing the animals and tissue resection, the effect of the delivered fibroblast growth factor, which stimulates angiogenesis, was assessed via the visual observation of blood around the implantation area and the hemoglobin count from the implanted tissues. The latter criteria were significantly higher in tissues treated with the growth factor loaded hydrogel [15].

The antibacterial efficacy of hydrogel drug delivery systems can be evaluated with mice in vivo [226]. Subcutaneous injection of S. aureus created the infection on the mice. Three groups were compared: A control group untreated, a second group injected with the unloaded polysaccharide (carboxymethyl chitosan and oxidative dextran) hydrogel, and a third group injected with a ceftriaxone antibiotic-loaded polysaccharide hydrogel. The histology analysis of the resected tissues demonstrated an anti-infective effect of the polysaccharide hydrogel developed [226].

In the previously mentioned in vivo studies, the utilization of scores to grade tissue samples and of staining and immunohistochemistry staining presented the common limitation of depending on the subjective visual observation of the researcher. The use of histopathological scores to quantify the qualitative observation of an image is referred to as subjective quantification or semi-quantitative. In addition, there is a lack of gold standard in the protocols to quantify histological outcomes from an image [227]. Therefore, the results are inherently variable and unreliable. The solution to this limitation is developing automated image analysis that processes the images based on the same thresholds, hence providing an objective quantification while also reducing the image processing time [228,229].

**Figure 10:**
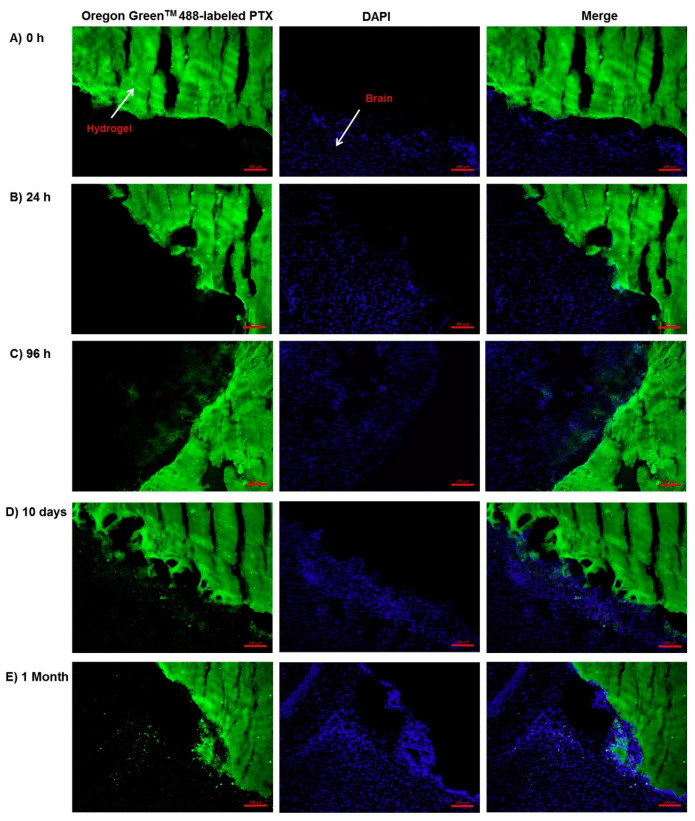
In Oregon Green™ 488-labeled paclitaxel (green) PEG-DMA hydrogels in healthy mouse brains. The local in vivo PTX diffusion was followed from (**A**) 0 h, and after (**B**) 24 h, (**C**) 96 h, (**D**) 10 days, and (**E**) 1 month. The cell nuclei (blue) were stained with DAPI. Scale bar = 100 μm (*n* = 3–6). Reproduced with permission from [45]. Copyright Elsevier, 2019.

Another discrepancy in the previously mentioned in vivo investigation is that the drug release is not accounted for nor reported. The direct assessment of the in vivo drug release should be monitored because it indicates the translatability of the in vitro results and predicts the drug pharmacokinetics in humans. In this respect, some researchers have performed elegant and meaningful correlations between in vitro and in vivo release. Zhao et al., for instance, demonstrated that the in vivo release of paclitaxel from their PLGA and polyethylene glycol dimethacrylate (PEG-DMA)-based hydrogel system designed to treat glioblastoma recurrence after surgical resection did not correlate with their previous in vitro release data [45,47]. The first in vitro study showed a burst release within the first day, followed by a sustained release that lasted 1 week. After 1 week, up to 28.9% of the drug cargo was released, but the limitation of the drug extraction method did not allow to measure further timepoints. However, the team expected the sustained release to last up to 4 weeks [47]. In their subsequent work, the paclitaxel in vivo release was evaluated qualitatively by fluorescence imaging (Figure 10). According to the fluorescence images of the hydrogel implant and surrounding brain tissue at implantation time (Figure 10A), after 24 h (Figure 10B), after 96 h (Figure 10C), after 10 days (Figure 10D), and after 1 month (Figure 10E) post-implantation, the in vivo release presented no burst release, and the paclitaxel was detected up to 1 month after implantation [45]. While the endeavors of the authors to correlate the in vitro and in vivo release of the drug released from the hydrogel drug delivery system should be noted, the quantification of the in vivo release was there qualitatively only.

A better method to provide quantitative measurement of the drug release is blood sample collection. Blood samples can be collected from animals at different time points to measure drug release. The blood collection was implemented in the design of a study to quantify the enteral release of vitamin C [12]. After collection, the drug was extracted from the blood samples via 30-minute coagulation followed by centrifugation, and the concentration was measured by a commercial kit based on the colorimetric Fast blue salt method [12]. Therein, researchers correlated the in vivo and in vitro release fractions and found close release percentages of vitamin C after 2 h, which is the time needed to pass the stomach, and after 6 h, when the vitamin C is in the small intestine and at the end of the release assay after 10 h (Figure 9F) [12]. Another way to detect plasmid (coding for the growth factor VEGF) released in vivo was to use RT-qPCR to detect and quantify expressed mRNA of VEGF and other inflammatory factors, IL-1β, TNF-α, from the harvested tissues. Compared to the groups not treated with the pDNA, the experimental group that was implanted with the hydrogel delivery system containing the pDNA led to a significant increase of VEGF expression in vivo. This assay could be considered as an indirect alternative to the evaluation of the release of pDNA. However, it was presented in relative RNA expression (in percentage) between experimental groups, which focuses more on the efficacy of the DDS than on the absolute quantity of pDNA delivered [10].

In vivo models represent a steppingstone in the development of a hydrogel drug delivery system. The highlighted studies, which correlated the in vivo drug release to their in vitro release, showed the direction for future investigations since correlating in vitro with in vivo is an important step for later clinical studies. While in vitro cell culture models allow for long-term and real-time monitoring of cell-cell interaction and response and behavior to the treatment and mechanisms [42], they are still limited because the environment, although increasingly sophisticated, does not represent the complexity of the human body. It is then that the in vivo approach provides the complementary cell-to-body interaction scale and the full complexity of the metabolism and immune system responding to the implantation of a hydrogel drug delivery system [223]. The in vitro cell culture models are essential to decipher fundamental cellular mechanisms and provide a proof of concept for the drug delivery system’s efficacy, which then needs to be tested in an in vivo model. The latter allows testing the devices and, most importantly, assess the translatability of the in vitro findings. Although indispensable, in vivo studies remain a significant time and financial investment and as well as require staff with the relevant expertise [230]. Finally, the automation of histology and immunohistochemistry stained tissue images should be implemented systematically to provide better reliability of the results.

## 6. Perspectives

Several review articles have highlighted the critical parameters governing drug release from hydrogel drug delivery systems. However, there was still a significant lack in the literature about the methodologies, specifically regarding the experimental release protocols, the interpretations of the release kinetics, and the evaluation of drug diffusion coefficients in hydrogel systems. As this was shown throughout several studies presented in this review, variability in any of these criteria alone can have a significant impact on the in vitro results and their ability to predict in vivo drug release behavior.

In this work, we reviewed the current protocols and techniques to quantify different types of drugs released, how to interpret drug release profiles, and how to measure the drug diffusion coefficient in hydrogels, aiming to provide guidelines for improved standardization of current practices in the hydrogel drug delivery field. The experimental conditions of in vitro release assays must be chosen to be the most physiologically relevant [33]. This means studying the release at physiological agitation, temperature, and pH. The volume of the release media must be maintained constant and ensure perfect sink conditions, and its composition must reflect physiological conditions. When hydrogel drug delivery systems are enzymatically degradable, drug release by hydrogel degradation should be performed to provide a more accurate prediction on in vivo release. Hu et al. designed an in vitro release of vitamin C with the previous considerations in mind that led to a precise prediction of in vivo release and should be taken as an example for the field [12]. They used appropriate simulated gastric fluid and simulated intestinal fluids, in which pH and composition (presence of enzyme) matched those of the oral administration route. In addition to mimicking the physiological environment, the fitting of cumulative release data with the Korsmeyer–Peppas, the zero-order, and the first order should be systematical to provide mechanistic insights into release kinetics and its tailoring [114]. Furthermore, including the ‘time’ parameter in the statistical analysis of cumulative release should be automatic in all systems, and thus we advise systemically using a general linear model for any statistical analysis of drug cumulative release curves [94]. Finally, the MODDE software allows statistical prediction of outputs parameters and thus circumvents the typical time-consuming trial-and-error investigations in use by several research groups.

Here, the evaluation techniques of the drug diffusion coefficient through hydrogels were also reviewed and shown to be valuable predictive characterization tools. They should be used more commonly and earlier in the development of hydrogel drug delivery for optimization purposes. For instance, preliminary studies should focus on characterizing the diffusivity properties of the hydrogels drug delivery system and the associated release kinetics to understand the potential of the hydrogel-drug delivery system fully in the first place. This approach may prove more beneficial than designing studies based on theoretical applications, which lack an accurate prediction of release behavior.

Finally, in vitro and in vivo models testing the efficacy of the drug release from hydrogel are the cornerstone of drug delivery system development to predict clinical outcomes. They should be complementary and represent the in vivo situation as close as possible, and may likely involve the use of 3D cell culture in vitro models that better represent the cellular context and in vivo models investigating both the release kinetics and drug effects. The automation of histology and immunohistochemistry staining images is the direction to take to improve the reliability of in vivo studies. Similarly, while presenting a complex undertaking, systematic drug detection, and quantification in vivo should be implemented as paramount to the translatability of the drug delivery system.

Ultimately, despite an array of techniques available, better use of those techniques and standardization in the analysis would strongly benefit the hydrogel-drug delivery field. Such an approach will leverage the versatile advantages of hydrogel-drug systems, in turn paving the way to meaningful systems being smartly designed and tested, and thus better and more quickly translated.

## Figures and Tables

**Table 1:** Advantages and disadvantages of drug quantification techniques. UV-Vis: Ultraviolet-Visible; ELISA: Enzyme-linked immunosorbent assay; HPLC: High-performance liquid chromatography; MS: Mass spectrometry; RT-qPCR: Real-time quantitative polymerase chain reaction. +: Low, ++: Medium, +++: High.

Criteria	UV-Vis Absorbance	Fluorescence	ELISA	HPLC-UV	HPLC MS	(RT)-qPCR
Convenience	+++	+++	+++	++	+	+
Low cost	+++	+++	++	++	+	+
Complexity	+	+	+	++	+++	+++
Sensitivity	+	++	++	++	+++	+++
Selectivity	+	++	+++	++	+++	+++

**Table 2:** Molecules delivered by hydrogel drug delivery systems and detected by UV-Vis spectrophotometry. N.G: Not given; N.A: Not applicable; Ref: reference.

Hydrogel Material(s)	Molecule	Molecule Function	Application	Wavelength of Detection (nm)	Ref
Oxidized dextran, gelatin, and hyaluronic acid	Naproxen	Anti-inflammatory	Tissue engineering	230	[7]
	Dexamethasone	Anti-inflammatory		243	
Silk sericin, polyvinyl alcohol	Gentamicin	Antibiotic	Wound dressing	335	[6]
	Aspirin	Anti-inflammatory		280	
Poloxamer 407	Methylene blue	Model molecule	Antimicrobial photodynamic therapy	664	[59]
Salecan, chitosan	Vitamin C	Nutrient	Food supplementation	265	[12]
N-succinyl chitosan-g-poly (acrylic acid)	Theophylline	Bronchodilator	Respiratory diseases	272	[60]
Gellan gum	Apigenin	Bronchodilator	Asthma	N.G.	[61]
2-Hydroxyethyl methacrylate, methyl methacrylate	Moxifloxacin	Antibiotic	Post-cataract removal prophylaxis	190 to 230	[27,62]
	Diclofenac	Anti-inflammatory			
	Levofloxacin	Antibiotic			
	Diclofenac	Anti-inflammatory			
	Ketorolac	Anti-inflammatory			
Carboxylated polyvinyl alcohol, gelatin, hyaluronic acid	Ampicillin	Antibiotic	Wound dressing	275	[63]
Chitosan, polyvinyl alcohol	Diflunisal	Anti-inflammatory	N.A.	252	[17]
Hyaluronic acid, dextran, β-cyclodextrin	Resveratrol	Anti-inflammatory	Wound healing	305	[10]
Xanthan	Pentoxifylline	Blood thinner	N.A.	274	[64]
Alginate polydopamine	Bortezomib	Anticancer	Cancer	270	[13]
Polyethylene glycol, polycaprolactone-triol	Diclofenac	Anti-inflammatory	Oral delivery	277	[16]
Linseed polysaccharide	Moxifloxacin	Antibiotic	Oral delivery	95	[65]
Hyaluronan, dextran	Nile red de	Model molecule	Diffusion model	N.G.	[66]
Chitosan, polyvinyl pyrrolidone	Cefixime	Antibiotic	N.A.	288	[67]
Alginate, carboxymethyl chitosan	Tetracycline hydrochloride	Hydrophilic Antibiotic	N.A.	363	[68]
	Silver sulfadiazine	Hydrophobic Antibiotic	N.A.	254	
Poly(N-isopropylacrylamide), N-tert-butylmaleimic acid	Rhodamine 6G	Model molecule	N.A.	527	[36]
Graphene, gelatin	Zoledronic acid	bisphosphonates	N.A.	N.G.	[69]
Anionic agarose-carbomer	Ibuprofen	Anti-inflammatory	Diffusion mechanism	264	[70]
Acrylamide-modified hyaluronic acid, folic acid, Fe^3+^	Acetylsalicylic acid (aspirin)	Analgesic	Wound dressing	N.G.	[19]
Scleroglucan	Theophylline	Bronchodilator	Drug diffusion model	271	[71]
DNA, oxidized alginate	Simvastatin	Local stem cell differentiation	Tissue engineering	240	[57]
Chitosan	Kartogenin	Cell differentiation	Cartilage tissue engineering	278.4	[72]

**Table 3:** Molecules delivered by hydrogel drug delivery systems and detected by fluorescence intensity. N.G: Not given; N.A: Not applicable; Ex: Excitation; Em: emission; Ref: reference; BSA: bovine serum albumin; FITC: luorescein-5-isothiocyanate; SYBR: Synergy Brands, Inc.

Hydrogel Material(s)	Molecule	Molecule Function	Application	Wavelength of Detection (nm)	Ref
Gelatin	Fluorescein	Model hydrophobic molecule	Tissue engineering	Ex: 485, Em: 535	[15]
Poly(ethylene glycol) dimethacrylate, poly lactic-co-glycolic acid	Paclitaxel-Oregon Green^TM^	Anticancer	Local cancer therapy	Ex: 488	[45]
Hyaluronan, alginate, gellan gum	Dextran-FITC	Diffusion model	Diffusion	Ex: 488, Em: 496–650	[89]
Hyaluronic acid, poly γ-glutamic acid	BSA-FITC	Model molecule	Cartilage tissue engineering	Ex: 493	[8]
Aptamer-tethered single-stranded DNA	Doxorubicin	Anticancer	Cancer	Em: 510	[90]
	SYBR Green I-dye	Model molecule	Cell imaging	Em: 497	
Carbon Dot, protoporphyrin IX, DNA	protoporphyrin IX	Antibacterial	Antibacterial photodynamic therapy	Em:410	[91]
DNA, Ag, agarose	Atto425 dye	Dye	Release model	Ex: 355, Em: 460	[92]
	Atto550 dye	Dye		Ex: 355, Em: 460	
Gelatin norbornene, poly(ethylene glycol) dithiol	microRNA	Cell differentiation (osteogenic)	Tissue engineering	N.G	[93]

**Table 4:** Experimental parameters for the in vitro release of molecules delivered by hydrogel drug delivery systems. PBS: Phosphate-Buffered Saline; SGF: Simulated gastric fluid; SIF: Simulated intestinal fluid; USP: United States Pharmacopoeia; BSA: Bovine serum albumin; N.G: Not given; N.A: Not applicable. Ref: reference.

Hydrogel Material(s)	Molecule	Release Media	Volume of Release Media (mL)	Sampled Volume Refreshed	Enzyme	pH	Temperature (°C)	Agitation (rpm)	Ref
Oxidized dextran, gelatin, and hyaluronic acid	Naproxen	PBS	10	No	No	7.4	37	No	[7]
	Dexamethasone								
Silk sericin, polyvinyl alcohol	Aspirin	PBS	N.G	No	No	7.4	37	No	[6]
	Gentamicin								
Poloxamer 407	Methylene blue	PBS	15	Yes	No	N.G	37	100	[59]
Salecan, chitosan	Vitamin C	SGF, SIF	50	Yes	Yes	1.2, 6.8	37	50	[12]
N-succinyl chitosan-g-poly (acrylic acid)	Theophylline	SGF, SIF	N.G	No	Yes	1.2, 7.4	37	No	[60]
Alginate, Chitosan	Resveratrol	HCl, PBS	100	No	No	1.2, 5.5, 6.8, 7.4	37	110	[102]
Gellan gum	Apigenin	PBS	50	No	No	1, 1.2, 2, 3, 4, 5, 6, 7, 7.4, 8	37	100	[61]
2-Hydroxyethyl methacrylate, methyl methacrylate	Moxifloxacin	PBS	15	No	No	N.G	20–24	150	[27,62]
	Diclofenac								
	Ketorolac								
	Levofloxacin								
Carboxylated polyvinyl alcohol, gelatin, hyaluronic acid	Ampicillin	N.G	N.G	Yes	No	7.4	37	No	[63]
Chitosan, polyvinyl alcohol	Diflunisal	PBS	50	Yes	No	7.4	RT	Yes	[17]
Hyaluronic acid, dextran, β-cyclodextrin	Resveratrol	PBS	20	Yes	No	N.G	N.G	No	[10]
Xanthan gum, silk fibroin, hyperbranched mushroom polysaccharide	BSA	PBS (0.02% of NaN_3_)	30	Yes	No	N.G	N.G	No	[58,118]
	5-fluorouracil								
Gelatin	Fluorescein	PBS	N.G	Yes	No	N.G	4, 37	Yes	[15]
Xanthan	Pentoxifylline	Pure H_2_O, HCl	900	No	No	1.2, 7	37 ± 0.5	50	[64]
Alginate polydopamine	Bortezomib	PBS	N.G	No	No	6.5, 7.4	N.G	No	[13]
Polyethylene glycol, polycaprolactone-triol	Diclofenac	HCl/KCl solution, PBS	10	Yes	No	1.6, 7.4	37.5	Yes	[16]
Linseed polysaccharide	Moxifloxacin	N.G (USP 37)	900	Yes	N.G?	1.2, 4.5, 6.8	37 ± 0.1	100	[65]
Hyaluronan, dextran	Nile red	Saline solution	5	No	No	N.G	25 ± 1	No	[66]
Chitosan, polyvinyl pyrrolidone	Cefixime	SGF	500	No	No	1.2	N.G	No	[67]
Alginate, carboxymethyl chitosan	Tetracycline hydrochloride	PBS	18	Yes	No	7.4	37	70	[68]
	Silver sulfadiazine								
Poly(N-isopropylacrylamide), N-tert-butylmaleimic acid	Rhodamine 6G	SGF, isotonic serum (NaCl), SIF	N.G	N.G	N.G?	1.2, 7, 7.2	23, 37	No	[36]
Graphene, gelatin	Zoledronic acid	PBS	N.G	Yes	No	N.G	37	No	[69]
Anionic agarose-carbomer	Ibuprofen	PBS	N.G	Yes	No	7.4	37	No	[70]
Acrylamide-modified hyaluronic acid, folic acid, Fe^3+^	acetylsalicylic acid (aspirin)	PBS	4	Yes	No	6.4, 7.4, 8.4	37	60	[19]
DNA, oxidized alginate	Simvastatin	PBS/Ethanol (1:1)	0.4	Yes	No	4.5	37	60	[57]
Chitosan	Kartogenin	PBS	2	Yes	No	7.4	37	No	[72]
Hyaluronic acid, poly γ-glutamic acid	BSA-FITC	PBS	10	Yes	No	7.4	37	Yes	[8]
Carbon Dot, protoporphyrin IX, DNA	protoporphyrin IX	PBS	N.G	Yes	No	7.4	37	No	[91]
Gelatin norbornene, poly(ethylene glycol) dithiol	microRNA	PBS	0.1	No	No	N.G	N.G	No	[93]
